# Challenge of Humans with Wild-type *Salmonella enterica* Serovar Typhi Elicits Changes in the Activation and Homing Characteristics of Mucosal-Associated Invariant T Cells

**DOI:** 10.3389/fimmu.2017.00398

**Published:** 2017-04-06

**Authors:** Rosângela Salerno-Goncalves, David Luo, Stephanie Fresnay, Laurence Magder, Thomas C. Darton, Claire Jones, Claire S. Waddington, Christoph J. Blohmke, Brian Angus, Myron M. Levine, Andrew J. Pollard, Marcelo B. Sztein

**Affiliations:** ^1^Center for Vaccine Development, University of Maryland School of Medicine, Baltimore, MD, USA; ^2^Department of Epidemiology and Public Health, University of Maryland School of Medicine, Baltimore, MD, USA; ^3^Oxford Vaccine Group, Department of Paediatrics, University of Oxford, Oxford, UK

**Keywords:** MAIT cells, T cells, human, gut, bacteria, *Salmonella*

## Abstract

Gastrointestinal infections by *Salmonella enterica* serovar Typhi (*S*. Typhi) are rare in industrialized countries. However, they remain a major public health problem in the developing world with an estimated 26.9 million new cases annually and significant mortality when untreated. Recently, we provided the first direct evidence that CD8^+^ MAIT cells are activated and have the potential to kill cells exposed to *S*. Typhi, and that these responses are dependent on bacterial load. However, MAIT cell kinetics and function during bacterial infections in humans remain largely unknown. In this study, we characterize the human CD8^+^ MAIT cell immune response to *S*. Typhi infection in subjects participating in a challenge clinical trial who received a low- or high dose of wild-type *S*. Typhi. We define the kinetics of CD8^+^ MAIT cells as well as their levels of activation, proliferation, exhaustion/apoptosis, and homing potential. Regardless of the dose, in volunteers resistant to infection (NoTD), the levels of CD8^+^ MAIT cells after *S*. Typhi challenge fluctuated around their baseline values (day 0). In contrast, volunteers susceptible to the development of typhoid disease (TD) exhibited a sharp decline in circulating MAIT cells during the development of typhoid fever. Interestingly, MAIT cells from low-dose TD volunteers had higher levels of CD38 coexpressing CCR9, CCR6, and Ki67 during the development of typhoid fever than high-dose TD volunteers. No substantial perturbations on the levels of these markers were observed in NoTD volunteers irrespective of the dose. In sum, we describe, for the first time, that exposure to an enteric bacterium, in this case *S*. Typhi, results in changes in MAIT cell activation, proliferation, and homing characteristics, suggesting that MAIT cells are an important component of the human host response to bacterial infection.

## Introduction

MAIT cells are an “innate” like population of T cells that display a TCR Vα7.2^+^CD161^+^ phenotype and are restricted by the non-classical MHC-related molecule 1 (MR1) (1–3). While thymic MAIT cells have the ability to respond to bacterial stimulation by upregulating activation (CD127) and proliferation (Ki67) markers, their cell surface phenotype is similar to that of naïve T cells (4, 5). After birth, MAIT cells acquire a memory phenotype and expand dramatically in the mucosa and periphery. MAIT cells are few and naive in cord blood (6) but constitute up to 10% of human blood T cells in adults (7), making their biology and behavior relatively easy to study in *ex vivo* assays. Human MAIT cells also express molecules such as CD26, CD45RO, and transcription factors RORγt and ZBTB16, which are involved in their function (7–10). Moreover, they express on their cell surface high levels of cytokine receptors for IL-18, IL-12, and IL-23 (6, 7) and can secrete TNF-α, IFN-γ, IL-17, granzyme a/b, and perforin (3, 7, 11, 12), the latter reinforcing their cytotoxic capability. MAIT cells can also harbor strong immune responses to various bacteria including gut commensals and pathogens from the *Enterobacteriaceae* family (e.g., *Salmonella*) (12). Recently, we provided the first direct evidence that CD8^+^ MAIT cells are activated, able to secrete cytokines and exhibit cytotoxic properties against B cells exposed to gut commensals and pathogens such as *Salmonella enterica* serovar Typhi (*S*. Typhi) (12). These responses were MR1 restricted and involved the endocytic pathway. Moreover, the quality of these responses (i.e., cytokine profiles) was dependent on bacterial load, but not on the level of expression of MR1 or bacterial antigen on B cell surfaces (12). Thus, the evaluation of the influence of the bacterial load in CD8^+^ MAIT cell kinetics may provide further insight of the CD8^+^ MAIT cell role during bacterial infection.

Challenge studies in which healthy adult volunteers are intentionally infected with wild-type pathogens to test drugs and vaccines are a particularly relevant model for *Salmonella* infection. In the 1950s and 1970s, Dr. Theodore E. Woodward and Dr. Myron M. Levine of the University of Maryland in Baltimore conducted challenge studies with *S*. Typhi that led to the use of chloramphenicol in the treatment of patients with typhoid fever (13) and helped speed up the development of the Ty21a typhoid vaccine (14), the only oral live-attenuated vaccine licensed in the US (15). More recently, Dr. Pollard’s group [Oxford Vaccine Group (OVG), UK] has shown that by using smaller inoculums of virulent *S*. Typhi [~10^3^ or ~10^4^ colony-forming units (CFU)] administered following ingestion of a bicarbonate solution, the challenge can be performed safely, with attack rates in excess of 50% (14). In a previous study (12), we evaluated the impact of bacterial load on the restimulation of a “pre-formed pool” of CD8^+^ MAIT cells. The present studies extend these observations by studying CD8^+^ MAIT cell expansion and/or burst size *in vivo* after exposure to different *S*. Typhi bacterial loads. Using cells from volunteers participating in an Oxford challenge study who received a low- or high dose of wild-type *S*. Typhi, we investigated the *ex vivo* kinetics of CD8^+^ MAIT cell responses for up to 28 days after the challenge. We also defined CD8^+^ MAIT cell proliferation (Ki67), activation (CD38 and HLA-DR), exhaustion/apoptosis (CD57, caspase-3), and homing (CCR9 and CCR6) markers in mediating these responses. Regardless of the dose, in volunteers resistant to the infection (NoTD), the levels of CD8^+^MAIT cells after *S*. Typhi challenge fluctuated around their baseline values (day 0). In contrast, in volunteers susceptible to the development of typhoid disease (TD) we observed a sharp decline in circulating CD8^+^ MAIT cells during the development of typhoid fever. Interestingly, we found that TD volunteers exposed to low doses of wild-type *S*. Typhi (10^3^ CFU) had higher levels of CD8^+^ MAIT cells coexpressing CD38 and either CCR9, CCR6, or Ki67 during the development of typhoid fever than TD volunteers receiving a high dose of *S*. Typhi (10^4^ CFU).

In sum, we describe, for the first time, that exposure to an enteric bacterium, in this case *S*. Typhi, results in changes in MAIT cell activation, proliferation, and homing characteristics. These results suggest that MAIT cells are an important component of the human host response to bacterial infection.

## Materials and Methods

### Subjects

Healthy volunteers, between 18 and 60 years old, were screened for good health by medical history, physical examination, and normal laboratory tests, including blood counts. Individuals who previously received typhoid vaccination or have a residence for >6 months in typhoid-endemic areas were not eligible to participate in the study. A 2-week course of antibiotic treatment was initiated if typhoid diagnosis occurred (temperature ≥38°C sustained ≥12 h or bacteremia) or at day 14 post-challenge for the volunteers who did not develop typhoid fever (14). Twenty volunteers, 10 volunteers who received high (10^4^ CFU) and 10 volunteers who received low (10^3^ CFU) dose of the *S*. Typhi inoculum, and whose peripheral blood mononuclear cells (PBMC) were available were selected for the studies described in this manuscript (Table S1 in Supplementary Material). High- and low-dose inoculum groups were composed of volunteers who developed TD or not (Table S1 in Supplementary Material). Time points selected for the studies described in this manuscript included before and up to 28 days after the challenge. PBMC isolated from these volunteers were studied *ex vivo* without any *in vitro* stimulation.

### Antibodies and Cell Culture Media

Cells were surface stained with anti-human monoclonal antibodies (mAbs) to CD3 (clone OKT3), CD14 (clone M5E2), CD19 (clone HIB19), CD161 (clone HP-3G10), TCR Vα7.2 (clone 3C10) (Biolegend, San Diego, CA, USA), CD4 (clone L200), CD8 (clone SK1), activated caspase-3 (clone C92-605), CCR6 (clone 11A9), HLA-DR (clone G46-6), Ki67 (clone B56) (BD Pharmingen, San Diego, CA, USA), CCR9 (clone 112509) (R&D, Minneapolis, MN, USA), CD38 (clone LS198.4.3) (Beckman-Coulter, Miami, FL, USA), and CD57 [clone TB01 (TB01); eBioscience, San Diego, CA, USA]. Antibodies conjugated to the following fluorochromes were used in these studies: fluorescein isothiocyanate (FITC), phycoerythrin (PE), peridinin chlorophyll protein (PerCP)-Cy5.5, PE-Cy7, energy coupled dye or PE-Texas-red conjugate (ECD), violet (V) 450 (e.g., similar to Pacific blue), brilliant violet (BV) 570, BV605, BV650, quantum dot (QD) 800, Alexa 647, allophycocyanin (APC)-Alexa 700 and APC-H7.

Culture medium consisted of RPMI 1640 (Gibco, Grand Island, NY, USA) supplemented with 100 U/ml penicillin, 100 µg/ml streptomycin, 50 µg/ml gentamicin, 2 mM l-glutamine, 2.5 mM sodium pyruvate, 10 mM HEPES buffer, and 10% heat-inactivated fetal bovine serum (R10).

### Surface and Intracellular Staining

*Ex vivo* PBMC were used for this experiment. Briefly, after overnight (16–18 h) resting at 37°C, 5% CO_2_, PBMC were harvested, stained with a dead-cell discriminator, yellow fluorescent viability dye (YEVID, Invitrogen, Carlsbad, CA, USA) (16), followed by surface staining with mAbs against caspase-3, CCR6, CCR9, CD3, CD4, CD8, CD14, CD19, CD38, CD57, CD161, HLA-DR, and TCRα 7.2 surface antigens and fixation and permeabilization with Fix & Perm cell buffers (Invitrogen, Carlsbad, CA, USA) (12, 16). Cells were then stained intracellularly for Ki67. Finally, cells were resuspended in fixation buffer (1% formaldehyde) and analyzed as soon as possible by flow cytometry on an LSR-II instrument (BD Biosciences). Data were analyzed with WinList v6.0 (Verity Software House, Topsham, ME, USA). Lymphocytes were gated based on their scatter characteristics. Single lymphocytes were gated based on forward scatter height vs. forward scatter area. A “dump” channel was used to eliminate dead cells (YEVID^+^) as well as macrophages/monocytes (CD14^+^) and B lymphocytes (CD19^+^) from analysis. This was followed by additional gating on CD3, CD8, CD161, and TCR Vα7.2 to identify MAIT cells. During sample acquisition, routinely 300,000–500,000 events were collected in the forward and side scatter lymphocyte gate. This large number of gated MAIT cell events was essential to ensure that a sufficient number of positive cells for defined subsets would be collected for each tube analyzed.

### Statistical Analysis

All statistical tests were performed using SAS 9.3 (Cary, NC, USA). Observations were grouped by day following challenge in the following periods: pre-challenge, days 1–4, days 7–9 or within 48–96 h of disease onset, and days 14–28. Volunteers generally contributed more than one observation to each time period. To compare mean values by time period and group, while accounting for correlation between multiple measures from the same volunteer at the same time period and across time periods, we used mixed effects models. These models, which include a random effect for the subject, were fit by restricted maximum likelihood. Correlations used the Pearson product–moment tests. *P* values <0.05 were considered significant.

## Results

### Kinetics of MAIT Cells over a 28-Day Post-Challenge Follow-Up

**Figure 1:**
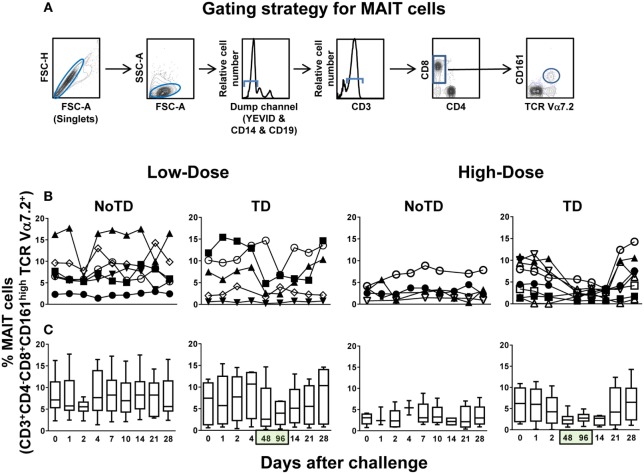
**Kinetics of MAIT cells over a 28-day post-challenge follow-up period**. *Ex vivo* peripheral blood mononuclear cells were stained with YEVID, followed by surface staining with monoclonal antibodies to CD3, CD4, CD8, CD14, CD19, CD161, and TCRα 7.2 and analyzed by multichromatic flow cytometry. For the analysis, following the elimination of doublets and other debris, the cells were gated on lymphocytes, and then a “dump” channel was used to eliminate dead cells (YEVID^+^) as well as macrophages (CD14^+^), and B cells (CD19^+^) from the analyses. This was followed by additional gating on CD3, CD4, and CD8, as well as CD161 vs. TCRα 7.2 to analyze MAIT cells. **(A)** A representative gating strategy for MAIT cells. **(B)** Individual volunteers and **(C)** combined data of MAIT cell kinetics. Bar graphs extend from the 25th to 75th percentiles, and the line in the middle represents the median of the pooled data from all the volunteers. The whiskers delineate the smallest to the largest value. Numbers in the “*X*” axis represent days after the challenge, except for the numbers inside of the green box that represent 48 and 96 h after diagnosis of typhoid disease. NoTD, volunteers who did not develop typhoid disease; TD, volunteers who developed typhoid disease.

Because of the potential importance of CD8^+^ MAIT cells (henceforth called MAIT cells) in resistance to bacterial infection, in particular to *Salmonella* infection (12), we investigated their kinetics in subjects participating in a dose-escalation challenge clinical trial conducted by Dr. Pollard’s group (Oxford Vaccine Group) (14). This study was performed using the antibiotic susceptible, virulent wild-type *S*. Typhi Quailes strain, which was first isolated in 1958 from the gallbladder of a known chronic carrier (14, 15, 17). Two separate cohorts were recruited, with one cohort receiving high (~10^4^ CFU) and another one receiving low (~10^3^ CFU) dose of the *S*. Typhi inoculum. The characteristics of the volunteers used for the studies presented in this manuscript are included in the Table S1 in Supplementary Material. Based on our previous data showing that *S*. Typhi-specific CD8^+^ T cell responses can be detected as early as 4 days after typhoid fever immunization (18, 19) or challenge with *S*. Typhi (20), we hypothesized that changes in MAIT cell kinetics might be seen at even earlier time points (e.g., days 1 and 2) after the challenge. Innate-like T cells such as MAIT cells are known to respond very rapidly (<2 h) after activation and within the first few days postinfection (5, 21). To test this hypothesis, *ex vivo* PBMC collected before and up to 28 days after the challenge (including days 1 and 2) were surface stained with mAbs to CD3, CD4, CD8, CD14, CD19, CD161, and TCRα 7.2 and analyzed by multichromatic flow cytometry. MAIT cells were defined as CD3^+^CD4^−^CD8^+^TCR Vα7.2^+^CD161^+^ cells (Figure 1A; Figure S1 in Supplementary Material). We found that regardless of the dose, in volunteers resistant to the infection (NoTD), the levels of MAIT cells after *S*. Typhi challenge fluctuated around their baseline values (day 0) (Figures 1B,C; Figures S2A and S3A in Supplementary Material). In contrast, in volunteers susceptible to the development of TD, we observed a sharp decline of MAIT cells 48 and 96 h after diagnosis, a time frame used to capture the events occurring immediately after the development of typhoid fever (Figures 1B,C; Figures S2A, S3A, and S4A in Supplementary Material). These results indicate that TD affects the kinetics of circulating MAIT cells. Of note, it is important to observe that the time to clinical diagnosis of typhoid fever was variable among the TD volunteers (i.e., ranging 6–13 and 6–10 days after the challenge for low- and high-dose volunteers, respectively) (Table S1 in Supplementary Material). For this reason, to align the time frames in which the various volunteers developed TD, we expressed the data as 48 and 96 h after typhoid diagnosis.

### MAIT Cell Activation following Challenge with Wild-type *S*. Typhi

**Figure 2:**
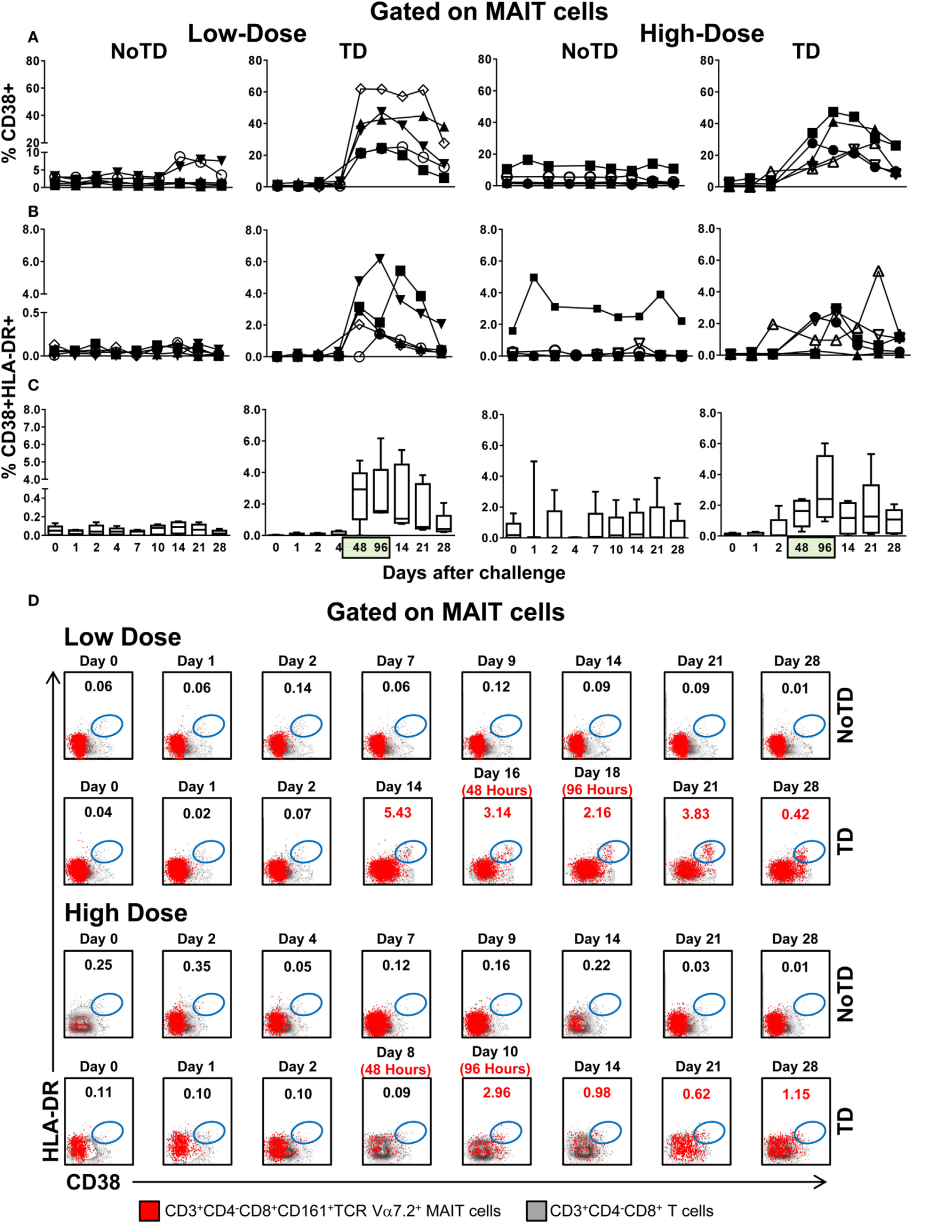
**MAIT cell activation following *Salmonella enterica* serovar Typhi challenge**. *Ex vivo* peripheral blood mononuclear cells were analyzed as described in Figure 1. Expression of CD38 and HLA-DR was evaluated to identify activated MAIT cells. Activated MAIT cells in individual volunteers expressing **(A)** CD38 or coexpressing **(B)** CD38 and HLA-DR surface markers. **(C)** Combined data of the activated MAIT cells coexpressing CD38 and HLA-DR surface markers in all volunteers. **(D)** Representative data of MAIT cells coexpressing CD38 and HLA-DR surface markers within the CD3^+^CD4^−^CD8^+^ T cell population. Bar graphs extend from the 25th to 75th percentiles, and the line in the middle represents the median of the pooled data from the five different subjects. The whiskers delineate the smallest to the largest value. Numbers in the “*X*” axis represent days after the challenge, except for the numbers inside of the green box that represent 48 and 96 h after diagnosis of typhoid disease. NoTD, volunteers who did not develop typhoid disease; TD, volunteers who developed typhoid disease.

To further investigate the association between decreases in the numbers of circulating MAIT cells and their functionality, we next measured the expression of CD38 and HLA-DR activation markers. CD38 and HLA-DR are well-known activation molecules expressed on the cell surface of activated T cells during the acute phase of bacterial and viral infections in humans (22–24). Regardless of the dose, we found that challenge elicited increased levels of activated MAIT cells (either CD38^+^ single positive or CD38^+^HLA-DR^+^ double positive) in TD volunteers, a phenomenon that was associated with the time of TD (48 and 96 h after diagnosis of typhoid fever) (Figures 2A–D). Interestingly, the magnitude of the activation of CD38^+^ single positive, but not CD38^+^HLA-DR^+^ double positive MAIT cells was significantly higher in volunteers in the low-dose cohort than in those in the high-dose group (Figure S4B in Supplementary Material). Only minimal perturbations on levels of CD38 or HLA-DR-expressing MAIT cells were observed in NoTD volunteers (Figures 2A–D; Figures S2B and S3B in Supplementary Material). Comparisons between the levels of activated MAIT cells (single CD38^+^ or CD38^+^HLA-DR^+^) and the levels of total MAIT cells (CD3^+^CD4^−^CD8^+^CD161^high^TCR Vα7.2^+^) were performed using Pearson product–moment correlation. We found an inverse correlation between MAIT cell activation and MAIT cell decline (*R*^2^ = 0.1207; *P* = 0.0004 and *R*^2^ = 0.1679; *P* = 0.0002 for levels of total MAIT cells vs. either single CD38^+^ or double CD38^+^HLA-DR^+^, respectively) (Figure S5 in Supplementary Material). Thus, high levels of either CD38^+^ single positive or CD38^+^HLA-DR^+^ double positive-activated MAIT cells are associated with a decline of MAIT cells in circulation.

### MAIT Cell Exhaustion and Apoptosis following Challenge with Wild-type *S*. Typhi

**Figure 3:**
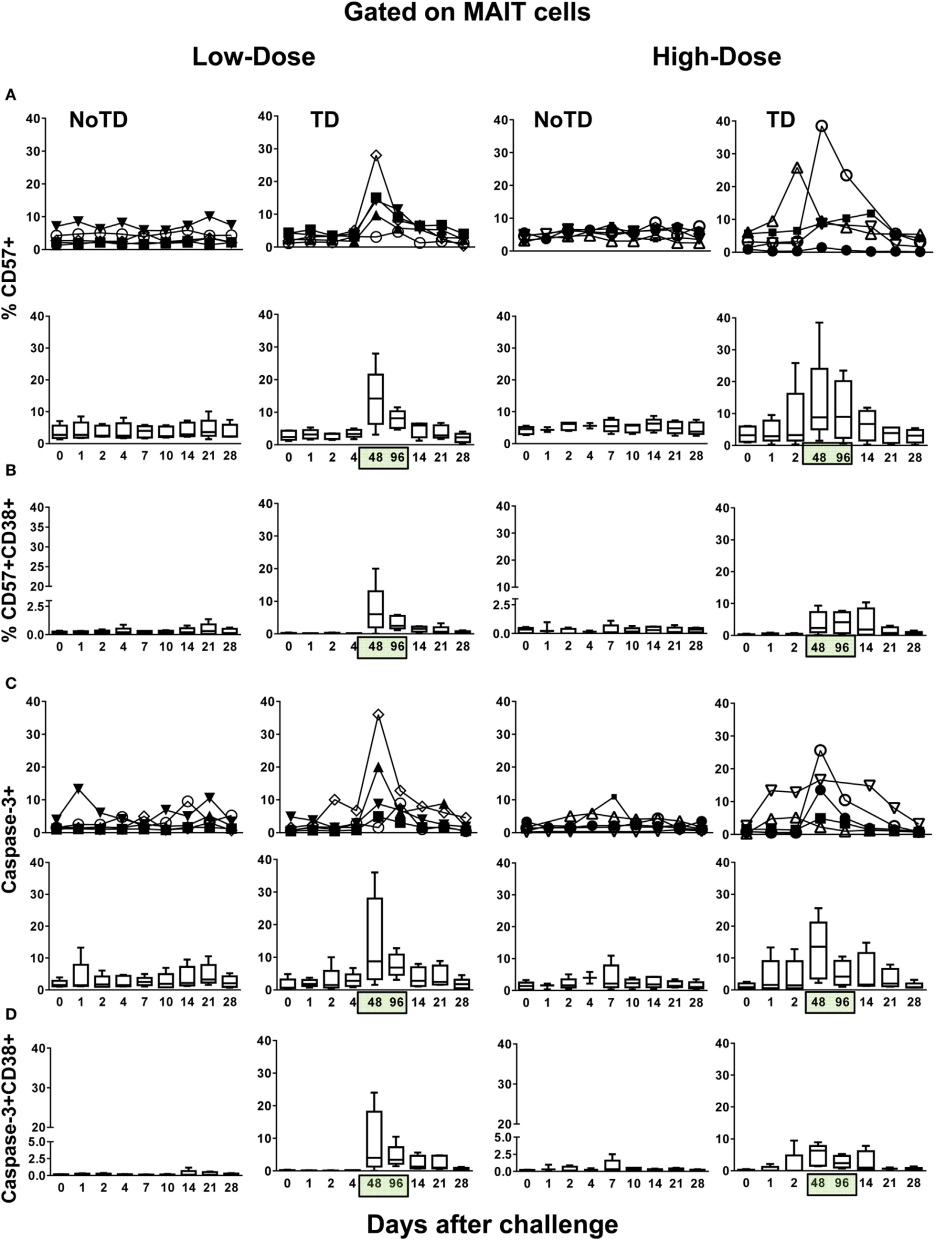
**Identification of exhausted and apoptotic MAIT cells following *Salmonella enterica* serovar Typhi challenge**. *Ex vivo* peripheral blood mononuclear cells were analyzed as described in Figure 1. Expression of CD57 and caspase-3 were performed to identify exhausted and apoptotic MAIT cells, respectively. Combined data of MAIT cells expressing **(A)** CD57 or coexpressing **(B)** CD57 and CD38 surface markers. Combined data of MAIT cells expressing **(C)** caspase-3 or coexpressing **(D)** caspase-3 and CD38 surface markers. Bar graphs extend from the 25th to 75th percentiles, and the line in the middle represents the median of the pooled data from the five different subjects. The whiskers delineate the smallest to the largest value. Numbers in the “*X*” axis represent days after the challenge, except for the numbers inside of the green box that represent 48 and 96 h after diagnosis of typhoid disease. NoTD, volunteers who did not develop typhoid disease; TD, volunteers who developed typhoid disease.

To provide additional insights into the decline of MAIT cells in circulation, we measured the expression of CD57, a molecule whose expression is associated with cell exhaustion (25), and caspase-3, a molecule indicative of apoptosis (26). Similar to the expression of CD38 and HLA-DR markers and regardless of the dose, we found high levels of MAIT cells expressing CD57 and caspase-3 markers in TD volunteers after typhoid fever diagnosis (Figures 3A,C; Figures S6–S9 in Supplementary Material). No significant differences were found in the levels of MAIT cells expressing either CD57 or caspase-3 between the high-dose and low-dose groups (Figure S10 in Supplementary Material). We also observed that the majority of the CD57 and caspase-3-expressing MAIT cells from TD participants were activated (Figures 3B,D; Figures S8 and S9 in Supplementary Material), and there was a direct correlation between the coexpression of CD38^+^HLA-DR^+^ and CD57^+^ (*R*^2^ = 0.1886; *P* = < 0.0001) and caspase-3^+^ (*R*^2^ = 0.1919; *P* < 0.0001) surface markers (Figure S11 in Supplementary Material). No substantial increases in CD57 or caspase-3 markers were observed in NoTD volunteers. These findings are consistent with a previously proposed model (27) in which MAIT cells are activated, exhausted, and depleted during the early stages of infection.

### MAIT Cell Proliferation Patterns following Challenge with *S*. Typhi

**Figure 4:**
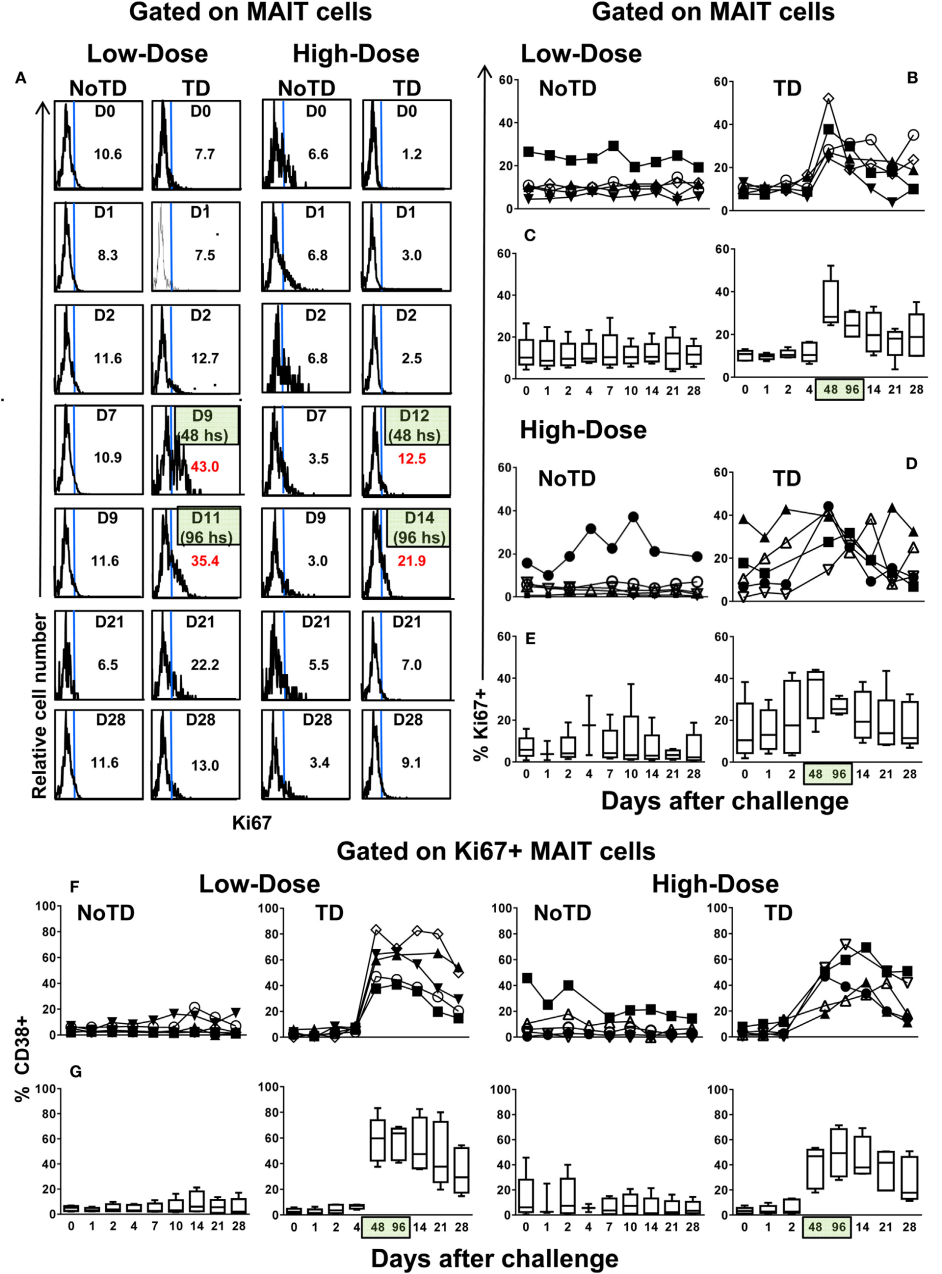
**MAIT cell proliferation following *Salmonella enterica* serovar Typhi challenge**. *Ex vivo* peripheral blood mononuclear cells were analyzed as described in Figure 1. Ki67 expression was used to identify proliferating MAIT cells. **(A)** Representative data of intracellular expression of Ki67^+^ MAIT cells over a 28-day post-challenge follow-up period in NoTD and typhoid disease (TD) volunteers who received a low- or high-dose challenge. **(B)** Individual and **(C)** combined data of proliferating MAIT cells from volunteers receiving a low-dose challenge. **(D)** Individual and **(E)** combined data of proliferating MAIT cells from volunteers receiving a high-dose challenge. **(F)** Individual and **(G)** combined data of CD38-positive cells gated on proliferating (Ki67^+^) MAIT cells. Bar graphs extend from the 25th to 75th percentiles, and the line in the middle represents the median of the pooled data from the five different subjects. The whiskers delineate the smallest to the largest value. Numbers in the “*X*” axis represent days after the challenge, except for the numbers inside of the green box that represent 48 and 96 h after diagnosis of typhoid disease. NoTD, volunteers who did not develop typhoid disease; TD, volunteers who developed typhoid disease.

**Figure 5:**
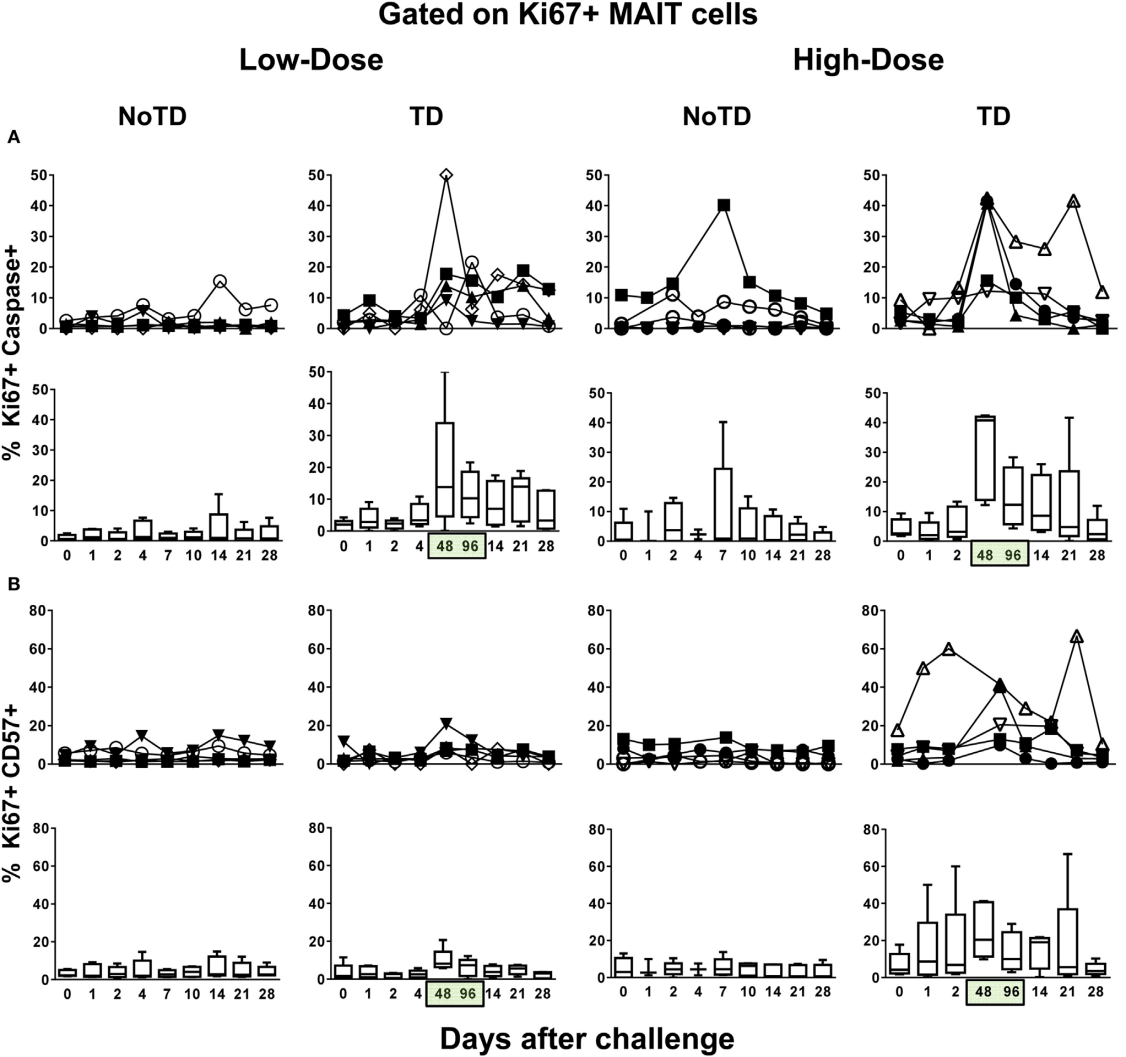
**Evaluation of exhaustion and apoptosis in proliferating MAIT cells**. *Ex vivo* peripheral blood mononuclear cells were analyzed as described in Figure 1. Expression of CD57 and caspase-3 were performed to identify exhausted and apoptotic in proliferating MAIT cells, respectively. Individual and combined data of Ki67^+^ MAIT cells expressing **(A)** capsase-3 or **(B)** CD57 surface markers are shown. MAIT cells were sequentially gated on Ki67^+^ and then on capsase-3 or CD57 surface markers. Bar graphs extend from the 25th to 75th percentiles, and the line in the middle represents the median of the pooled data from the five different subjects. The whiskers delineate the smallest to the largest value. Numbers in the “*X*” axis represent days after the challenge, except for the numbers inside of the green box that represent 48 and 96 h after diagnosis of typhoid disease. NoTD, volunteers who did not develop typhoid disease; TD, volunteers who developed typhoid disease.

Because a well-described consequence of immune activation is cell proliferation, we hypothesized that MAIT cells from TD volunteers might have been more susceptible to activation-induced proliferation than MAIT cells from NoTD volunteers. To test this hypothesis, we examined the levels of Ki67 proliferation-associated antigen on MAIT cells. We found that regardless of the dose, Ki67^+^ MAIT cells expanded after the challenge in TD volunteers, with sharp increases observed at 48 and 96 h after diagnosis of TD (Figures 4A–E; Figures S12A and S13A in Supplementary Material). Interestingly, while during the development of typhoid fever, the proliferation of CD38-positive MAIT cells was significantly higher in low-dose TD volunteers than in high-dose TD volunteers (Figures 4F,G; Figure S14B in Supplementary Material); no differences between the coexpression of Ki67 and either caspase or CD57^+^ were found between the high- and low-dose groups (Figures 5A,B; Figures S12B, S13B, and S14B in Supplementary Material).

### MAIT Cell Homing Patterns following Challenge with *S*. Typhi

**Figure 6:**
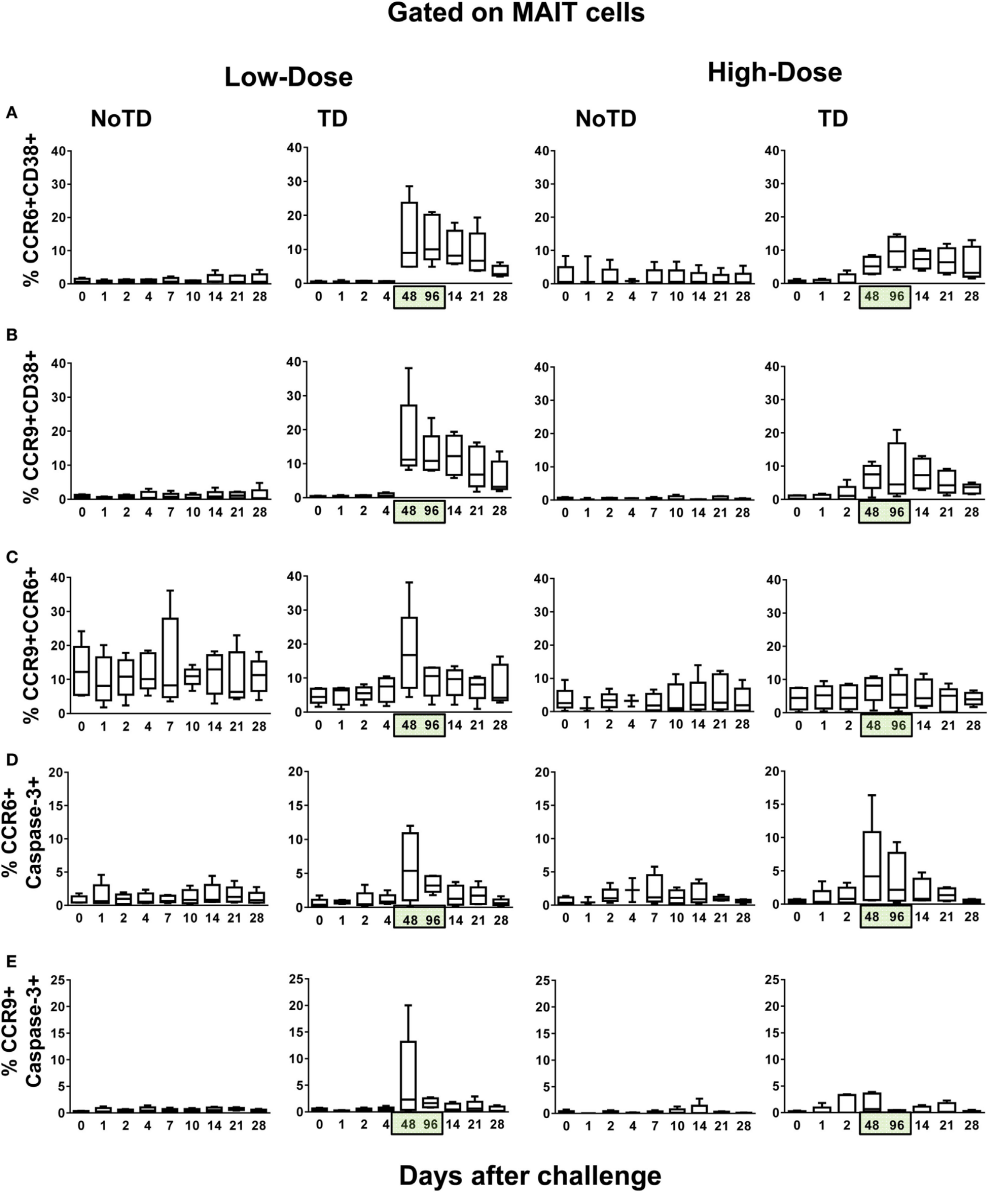
**Evaluation of the homing potential of MAIT cells following *Salmonella enterica* serovar Typhi challenge**. *Ex vivo* peripheral blood mononuclear cells were analyzed as described in Figure 1. Additional gating on CCR6 and CCR9 were performed to identify MAIT cells with the potential to home to inflamed and gut tissues, respectively. Combined data of MAIT cells coexpressing **(A)** CCR6 and CD38, **(B)** CCR9 and CD38, **(C)** CCR6 and CCR9, **(D)** CCR6 and caspase-3, and **(E)** CCR9 and caspase-3 surface markers. Bar graphs extend from the 25th to 75th percentiles, and the line in the middle represents the median of the pooled data from the five different subjects. The whiskers delineate the smallest to the largest value. Numbers in the “*X*” axis represent days after the challenge, except for the numbers inside of the green box that represent 48 and 96 h after diagnosis of typhoid disease. NoTD, volunteers who did not develop typhoid disease; TD, volunteers who developed typhoid disease.

An explanation for the lower numbers of activation-dependent MAIT cell proliferation detected in the blood of high-dose TD volunteers as compared with low-dose TD volunteers is that the MAIT cells from the former volunteers home earlier and/or at higher levels to other compartments in the host (e.g., gut and inflamed tissues). To address this possibility, we measured the expression of CCR9 and CCR6 molecules, which are involved in the homing of cells to the gut and inflamed tissues, respectively. We found that, during typhoid fever, although the frequencies of CD38^+^-activated MAIT cells expressing CCR6 or CCR9 homing markers were increased in all TD volunteers (regardless of the dose), their expression levels in the high-dose group were significantly lower than those observed in TD volunteers from the low-dose group (Figures 6A,B; Figures S6, S7, and S15–S17 in Supplementary Material). Moreover, the magnitude of MAIT cells coexpressing CCR6 and CCR9 markers were significantly higher, during typhoid fever, in TD volunteers receiving the low-dose inoculum than in TD volunteers receiving the high-dose inoculum (Figure 6C; Figures S6, S7, and S15–S17 in Supplementary Material). We also noted that the frequencies of CD38-positive MAIT cells expressing either CCR6 or CCR9 homing molecules remain unchanged in NoTD from both high- and low-dose groups (Figures 6A,B). Thus, the exposure to higher numbers of organisms might affect the homing of MAIT cells to inflamed tissues. To evaluate this possibility, we measured the presence of MAIT cells coexpressing caspase-3 and either CCR6 or CCR9 markers. We found that, although MAIT cells coexpressing caspase-3 and either CCR6 or CCR9 markers were significantly higher in TD (during typhoid fever) than in NoTD (7–9 days after challenge) volunteers, no significant differences were observed when comparing low- and high-dose TD volunteers at 48–96 h (Figures 6D,E; Figure S18 in Supplementary Material). The differences between TD and NoTD volunteers might be due to a disproportionate number of activation-induced proliferating MAIT cells undergoing caspase-3-mediated death during typhoid fever. On the other hand, the differences between TD volunteers receiving high and low doses might result from the numbers of MAIT cells recorded in circulation, which are homing to the inflamed gut, as measured by the expression of gut homing molecules.

## Discussion

Despite the predicted importance of MAIT cell responses in protection against bacterial infection, very limited data are available concerning the time kinetics and characterization of these responses following bacterial infection in humans. Here, we described for the first time the kinetics of MAIT cells up to 28 days following oral challenge of volunteers with wild-type *S*. Typhi. We observed that MAIT cells, possibly because of increased exposure *in vivo* to the microbes or their components, are activated, exhausted, and depleted during the infection.

MAIT cells are an important physiological component of the bacterial host defense and may also be involved in inflammatory disorders (7, 28, 29). In fact, previous studies have demonstrated that MAIT cells may play a significant role in *Vibrio cholerae* (30), *Mycobacterium tuberculosis* (TB) (31), and human immunodeficiency virus (HIV) (27, 32) infections in humans. For example, Gold and colleagues have shown that in humans, MAIT cells are decreased in the blood of patients with active TB infection (31). In other studies, levels of MAIT cells were found to be severely reduced in the circulation of patients with HIV-1 infection (27, 32), and their decline was associated with the time of diagnosis (27). Thus, we hypothesized that upon microbial stimulation by *S*. Typhi, functionally active circulating MAIT cells expressing gut migratory patterns might play a role in the *S*. Typhi infection.

In this manuscript, we reported that the levels of proliferating MAIT cells from TD volunteers were higher than in NoTD volunteers. These differences were not caused by altered levels of T-cell subsets at baseline (day 0), since baseline levels of CD3^+^, CD8^+^, and MAIT cell populations were similar among TD and NoTD volunteers (Figure S19 in Supplementary Material). A key property of protective immunity is the ability of conventional CD8^+^ T cells to undergo rapid proliferation, so that a large pool of memory cells is available to fight the infection (33). After birth, MAIT cells acquire a memory phenotype and expand dramatically in the mucosa and periphery (6). Thus, similar to conventional CD8^+^ T cells, MAIT cell expansion is likely to be the result of the antigen presentation. Thus, one would expect that increased bacterial loads would lead to greater MAIT cell proliferation. However, during typhoid fever, we found higher levels of activated (CD38^+^) proliferating MAIT cells in TD volunteers receiving a low dose than in those TD volunteers receiving a high dose of the inoculum. Thus, it is reasonable to speculate that this phenomenon might be the result of factors such as the initial amount of the inoculum and its ability to activate MAIT cell responses. This assumption is based on previous work from our group and others showing that the quality of MAIT cell responses is dependent on bacterial load (12), and that the accumulation of MAIT cells in the mucosa is dependent on the size of the bacterial inoculum (34). Previous observations showing that partial or incomplete antigenic activation can induce defects in proliferation (35) also support this theory. Moreover, previous reports had shown that MAIT cells are more susceptible to activation-driven apoptosis than conventional T cells (36), and that antigen-driven CD8^+^ T cell proliferation is lower in volunteers exhibiting high viral load (37, 38). However, it is not possible to exclude the possibility that the differential proliferation might be due to an increased level of MAIT cells undergoing apoptosis after being infected by *S*. Typhi. In fact, previous studies from our group have shown that *S*. Typhi readily infects T cells (39) and that heavily *S*. Typhi-infected cells can undergo apoptosis-mediated cell death (40, 41).

Our results also suggest that MAIT cells from TD volunteers acquire different homing patterns in function of the initial bacterial dose. As previously demonstrated in cells isolated from blood (7, 32) and colon (42), most MAIT cells express the chemokine receptors CCR6 and CCR9, presumably contributing to their intestinal localization. Here, we report that activated (CD38^+^) MAIT cells coexpressing CCR6 and CCR9 markers were significantly higher during typhoid fever in TD volunteers receiving the low-dose inoculum than in TD volunteers receiving the high-dose inoculum. Thus, increases of CCR6 and CCR9-positive MAIT cells coexpressing CD38 observed in circulation might translate to a lower recruitment of MAIT cells to the inflamed intestinal mucosa in volunteers receiving the high-dose inoculum. This hypothesis is consistent with recent work showing that MAIT cell numbers are lower in the intestinal mucosa of patients with inflammatory bowel disease than in healthy controls (43). However, little is known about the initial steps triggering MAIT cell homing to the inflamed tissues. Thus, studies aiming to examine the interactions between MAIT cells and antigen-presenting cells are needed to define further and characterize the effects of the inoculum size on MAIT cell homing behavior.

It is important to note that the investigators are acutely aware that the relatively small number of volunteers available for the studies presented in this manuscript, largely resulting from the limitations inherent in the performance of challenges with wild-type organisms in humans, is an overall weakness. However, this weakness will mainly affect the study if the null hypothesis is not rejected. Indeed, the relatively small number of volunteers studied did not preclude us from observing strong, statistically significant differences between volunteers allocated to the different cohorts. Upcoming studies with larger numbers of volunteers are required to confirm and expand the findings detailed in this manuscript.

In sum, to the best of our knowledge, this is the first body of work to show in humans the kinetics and characteristics of circulating MAIT cells after *S*. Typhi infection. These observations are of particular importance in that they provide direct evidence in humans that the exposure to *S*. Typhi not only alters the levels of activation of MAIT cells but also influences their proliferation and homing patterns. These findings also argue that MAIT cells play an integral role in the immune response against *Salmonella* infection and might help to maintain a balance between healthy and disease in the gut microenvironment. Gastrointestinal infections by *S*. Typhi are rare in industrialized countries. However, it remains a major public health problem in the developing world. Control measures such as sanitation, food hygiene, and vaccination are high priorities.

## Ethics Statement

All blood collection was approved by National Research Ethic Service (NRES), Oxfordshire Research Ethics Committee A (10/H0604/53). This protocol has been conducted in accordance with the ethical standards laid down in the 1964 Declaration of Helsinki and the principles of the International Conference on Harmonization Good Clinical Practice guidelines (14, 44). Volunteers were explained the purpose of this study and gave informed, signed consent before the blood draw. All blood specimens were processed within 4 h of the blood draw. PBMC were isolated from the blood by density gradient centrifugation and cryopreserved in liquid N_2_ following standard techniques (14).

## Author Contributions

RS-G designed the study, performed the experiments, analyzed the data, and wrote the manuscript; DL and SF performed the experiments, analyzed the data, and helped draft the manuscript; LM contributed to the design, performed statistical analyses, and helped draft the manuscript; TD, CJ, CW, CB, and BA contributed to the design, collected and processed the clinical samples, and helped draft the manuscript, ML developed the challenge model, contributed to the design, and helped draft the manuscript; AP and MS contributed to the design and analysis of the data and wrote the manuscript.

## Conflict of Interest Statement

The authors declare that the research was conducted in the absence of any commercial or financial relationships that could be construed as a potential conflict of interest.
