# Supplementary material for: Challenge of Humans with Wild-type Salmonella enterica Serovar Typhi Elicits Changes in the Activation and Homing Characteristics of Mucosal-Associated Invariant T Cells
Source: Front Immunol. 2017 Apr 6;8:398. doi: 10.3389/fimmu.2017.00398 (PMC5382150; doi:10.3389/fimmu.2017.00398)
Supplement: Supplementary file 1 [file presentation_1.pdf]

**Supplementary Table 1.** Demographics, challenge dose and clinical outcome of the participants included in this study

| | Low Dose | | High Dose | |
|---|---|---|---|---|
| Characteristics | NoTD* | TD** | NoTD | TD |
| Number of Participants | 6 | 5 | 5 | 8 |
| Age, mean (range) | 33 (21-46) | 26 (19-46) | 25 (19-35) | 30 (20-44) |
| Sex, # females (%) | 2 (33) | 0 (0) | 2 (40) | 3 (37) |
| *S*. Typhi challenge dose , mean (range) | $1.2 \times 10^3$ ($0.7\text{-}1.8 \times 10^3$) | $1.4 \times 10^3$ ($1.0\text{-}1.8 \times 10^3$) | $2.2 \times 10^4$ ($1.9\text{-}2.7 \times 10^4$) | $2.0 \times 10^4$ ($1.5\text{-}2.7 \times 10^4$) |
| Time to clinical Diagnosis, day, mean (range) | - | 8 (6-13) | - | 8 (6-10) |

*NoTD,individuals who did not develop typhoid disease

**TD, individuals who developed typhoid disease

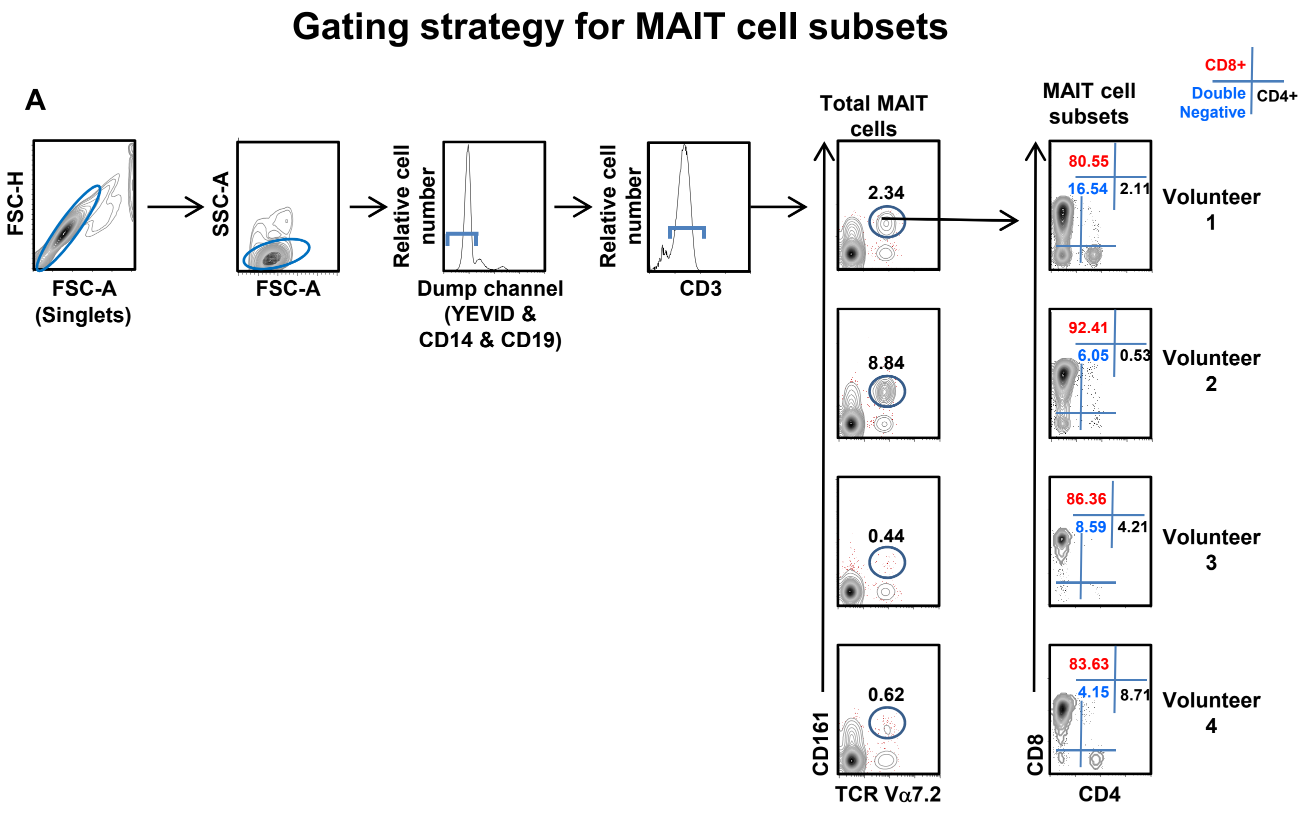


**Supplementary Figure 1. Amount of CD8⁺ subsets among total MAIT cells**. *Ex vivo* were stained with YEVID, followed by surface staining with mAbs to CD3, CD4, CD8, CD14, CD19, CD161 and TCRα 7.2, and analyzed by multichromatic flow cytometry. For the analysis, following the elimination of doublets and other debris, the cells were gated on lymphocytes, and then a "dump" channel was used to eliminate dead cells (YEVID⁺) as well as macrophages (CD14⁺), and B cells (CD19⁺) from the analyses. This was followed by additional gating on CD3 and CD161 versus TCRα 7.2 to analyze total MAIT cells. Amount of CD8 MAIT cell subsets was detected by additional gating on CD4 and CD8. Numbers represent the % positive cells in each quadrant.

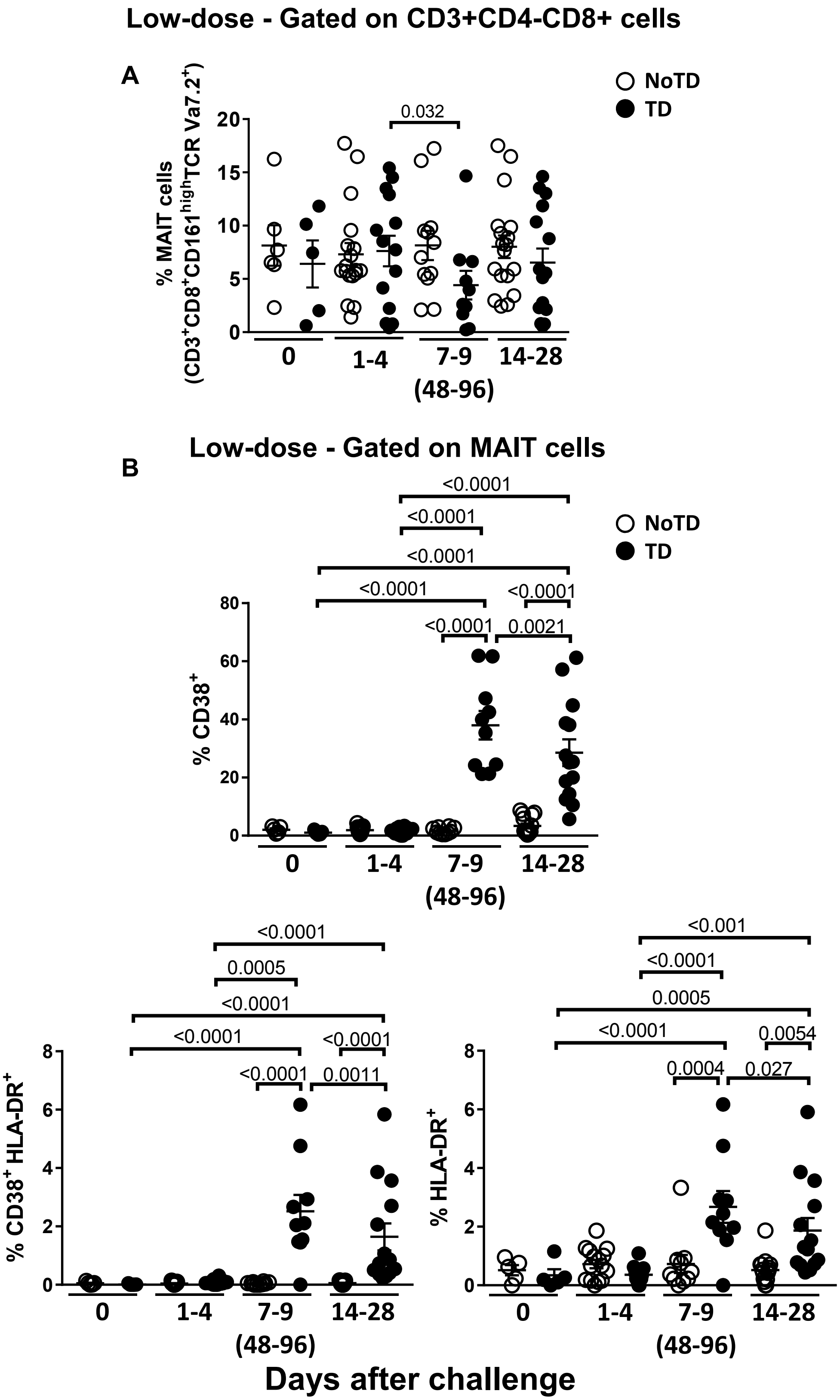


**Supplementary Figure 2. Comparison of the kinetics and activation markers on MAIT cells among volunteers receiving a low-dose inoculum**. *Ex vivo* PBMC isolated before (Day 0, baseline) and after challenge were analyzed as described in Figure 1. Levels of (A) total MAIT cells or (B) MAIT cells co-expressing CD38 and/or HLA-DR surface markers were evaluated and compared among NoTD and TD volunteers who received a low-dose inoculum. Data was grouped by time frames as follows: day 0, days 1-4, days 7-9 (for NoTD, or 48-96 hours for TD volunteers) and days 14-28. NoTD, individuals who did not develop typhoid disease. TD, Individuals who developed typhoid disease. *P* values of <0.05 were considered statistically significant.

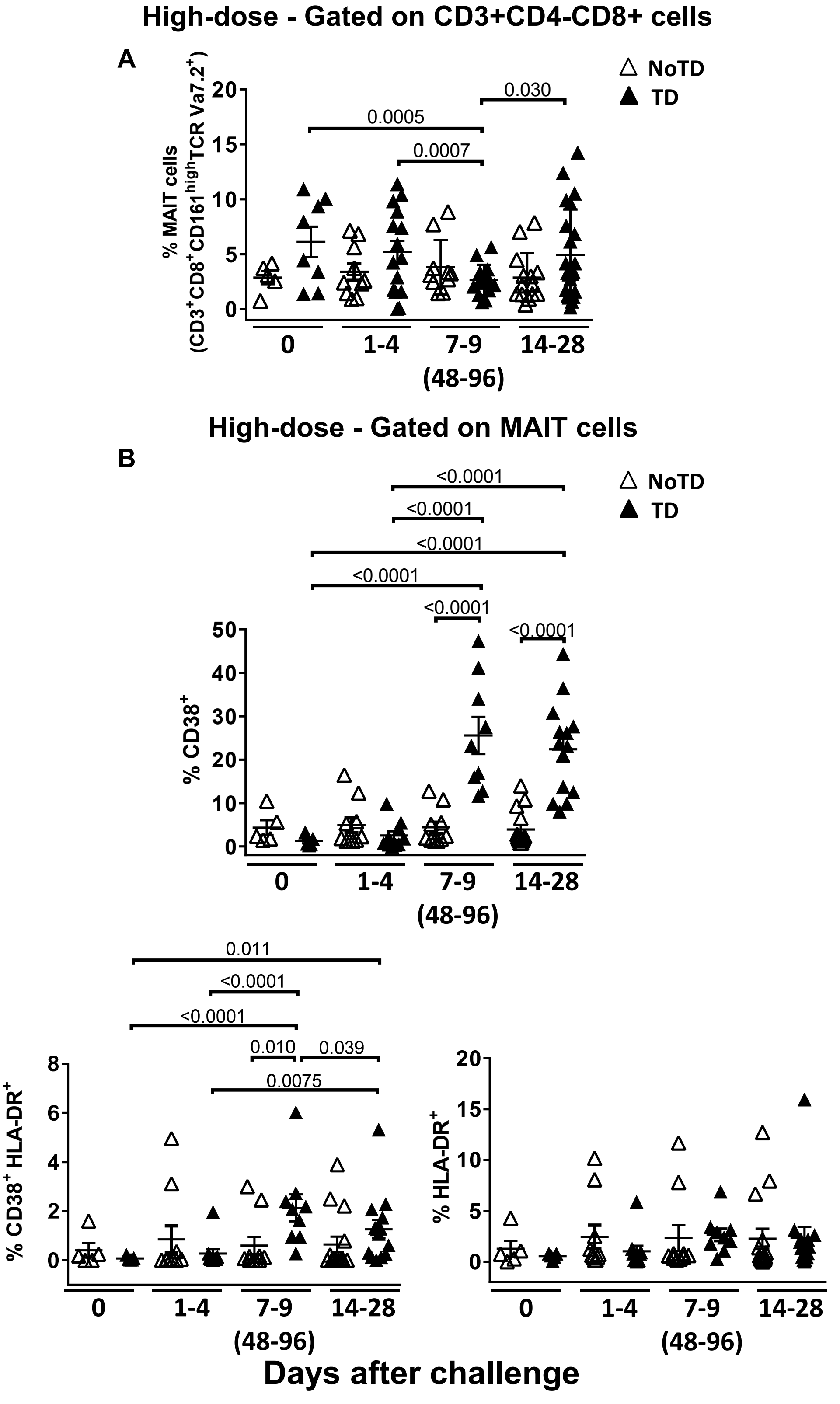


**Supplementary Figure 3. Comparison of the kinetics and activation markers on MAIT cells among volunteers receiving a high-dose inoculum**. *Ex vivo* PBMC isolated before (Day 0, baseline) and after challenge were analyzed as described in Figure 1. Levels of (A) total MAIT cells or (B) MAIT cells co-expressing CD38 and/or HLA-DR surface markers were evaluated and compared among NoTD and TD volunteers who received a high-dose inoculum. Data was group by time frame as follows: day 0, days 1-4, days 7-9 (for NoTD, or 48-96 hours for TD volunteers) and days 14-28. NoTD, individuals who did not develop typhoid disease. TD, Individuals who developed typhoid disease. *P* values of <0.05 were considered statistically significant.

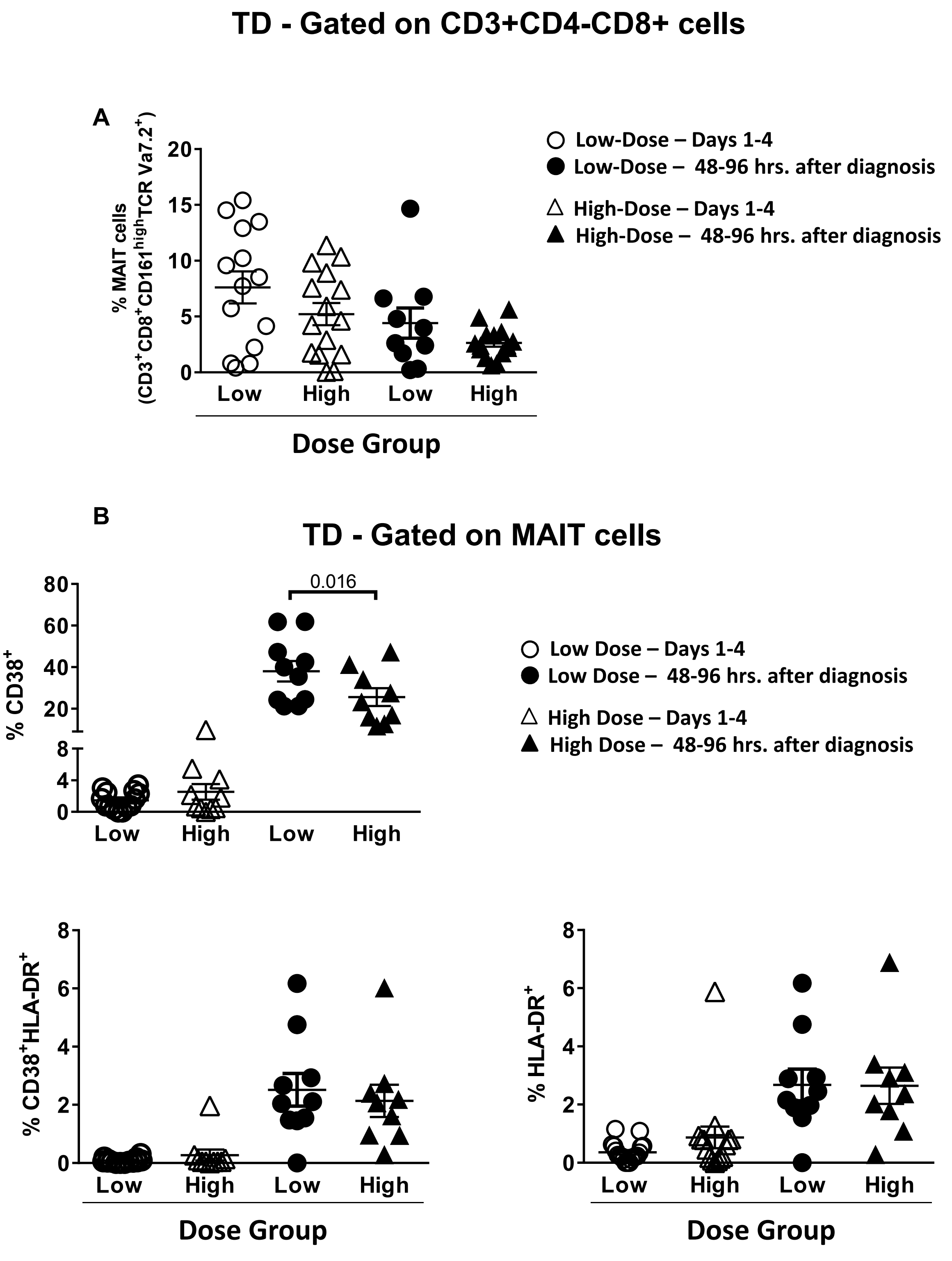


**Supplementary Figure 4. Comparison of the kinetics and activation markers on MAIT cells among TD volunteers receiving high and low-dose inoculum**. *Ex vivo* PBMC isolated before (Day 0, baseline) and after challenge were analyzed as described in Figure 1. Levels of (A) total MAIT cells or (B) MAIT cells co-expressing CD38 and/or HLA-DR surface markers were evaluated and compared among TD volunteers receiving a high or low-dose inoculum. Data was grouped by time frame as follows: before (days 1-4) and after development of disease (48-96 hours after diagnosis. TD, Individuals who developed typhoid disease. *P* values of <0.05 were considered statistically significant.

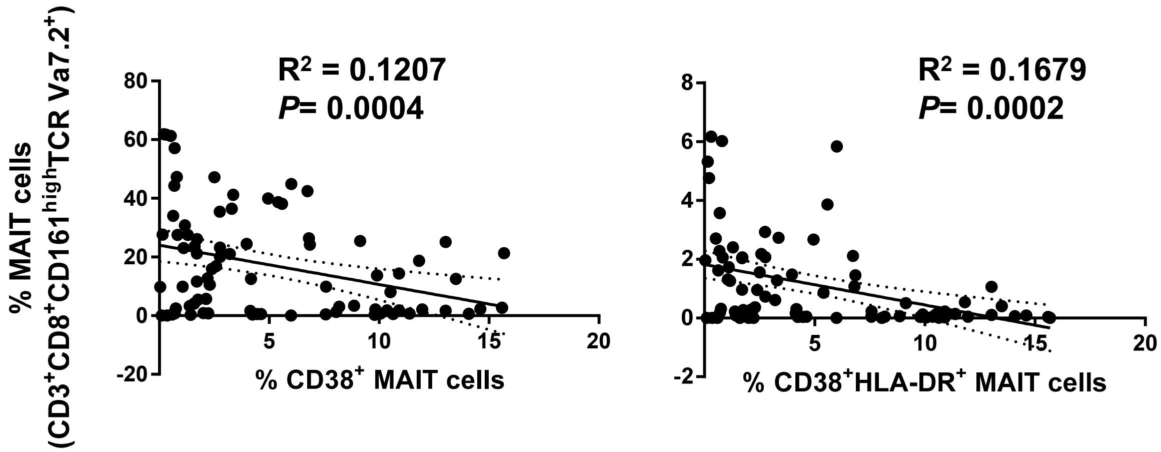


**Supplementary Figure 5. Inverse correlation between MAIT cell activation and MAIT cell decline**. *Ex vivo* PBMC were analyzed as described in Figures 1 & 2. The level of activated MAIT cells (either CD38+ single positive or CD38+HLA-DR+ double positive) was correlated to the levels of total MAIT cells (CD3+CD4-CD8+CD161highTCR Vα7.2+) in specimens from TD individuals collected before and up to 28 days after challenge. Coefficients of determination "R2" and the "*P*" values are shown. *P* values of <0.05 were considered statistically significant.

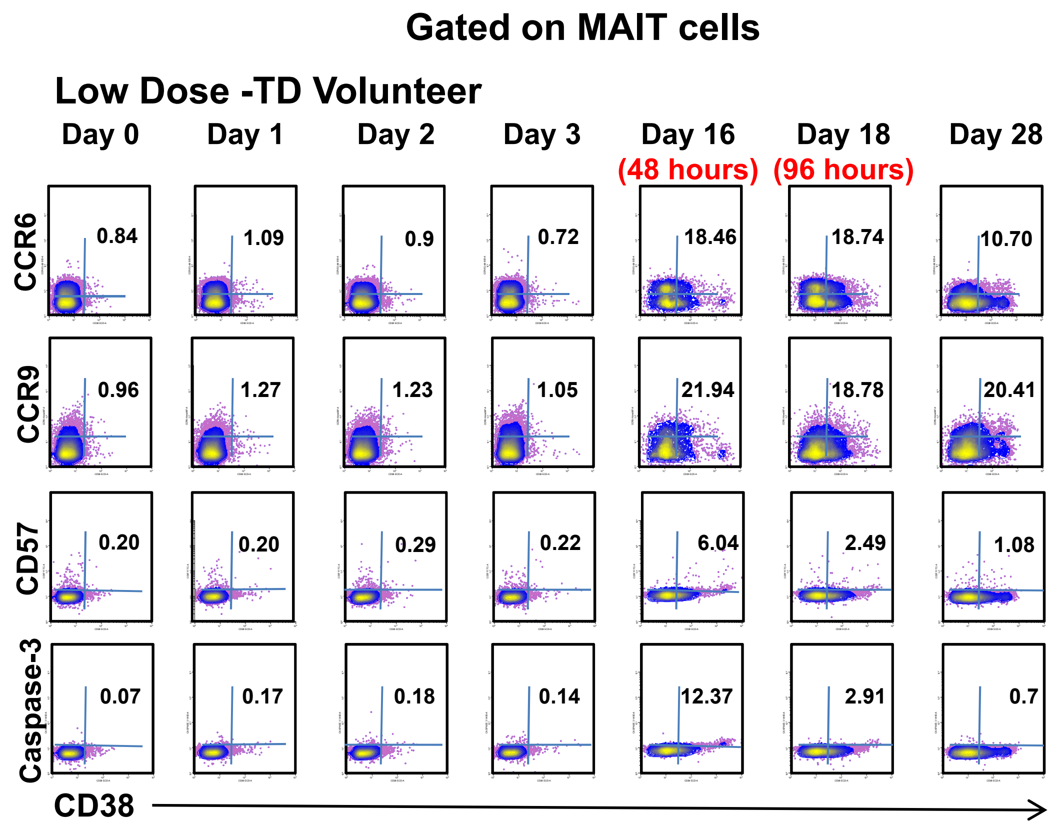


**Supplementary Figure 6. Analysis of CD38 expression on MAIT cells from a representative TD volunteer receiving a low-dose inoculum**. *Ex vivo* PBMC collected before (Day 0, baseline) and after challenge (days 1, 2, 3, 16 [48 hours after diagnosis], 18 [96 hours after diagnosis], and 28) were analyzed as described in Figure 1. Shown is additional gating on CD38 versus CCR6, CCR9, CD57 and Caspase-3. Numbers represent the % positive cells in the double-positive quadrant.

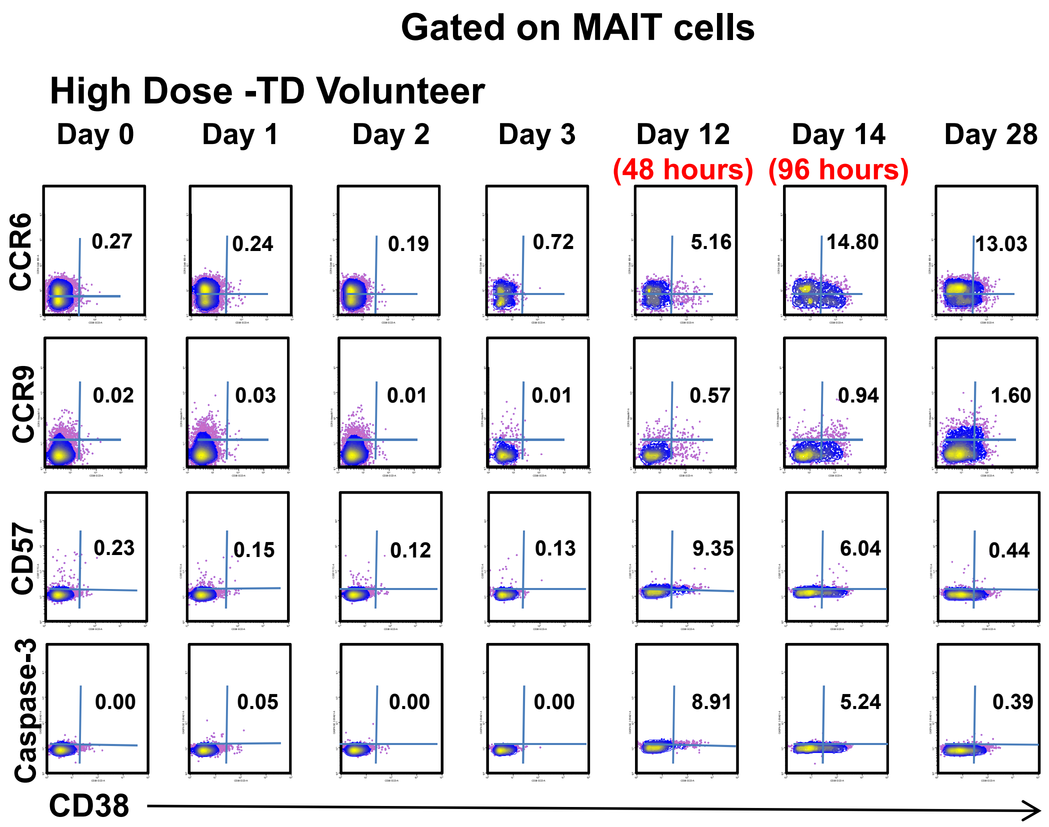


**Supplementary Figure 7. Analysis of the CD38 expression on MAIT cells from a representative TD volunteer receiving a high-dose inoculum**. *Ex vivo* PBMC collected before (Day 0, baseline) and after challenge (days 1, 2, 3, 12 [48 hours after diagnosis], 14 [96 hours after diagnosis], and 28) were analyzed as described in Figure 1. Shown is additional gating on CD38 versus CCR6, CCR9, CD57 and Caspase-3. Numbers represent the % positive cells in the double-positive quadrant.

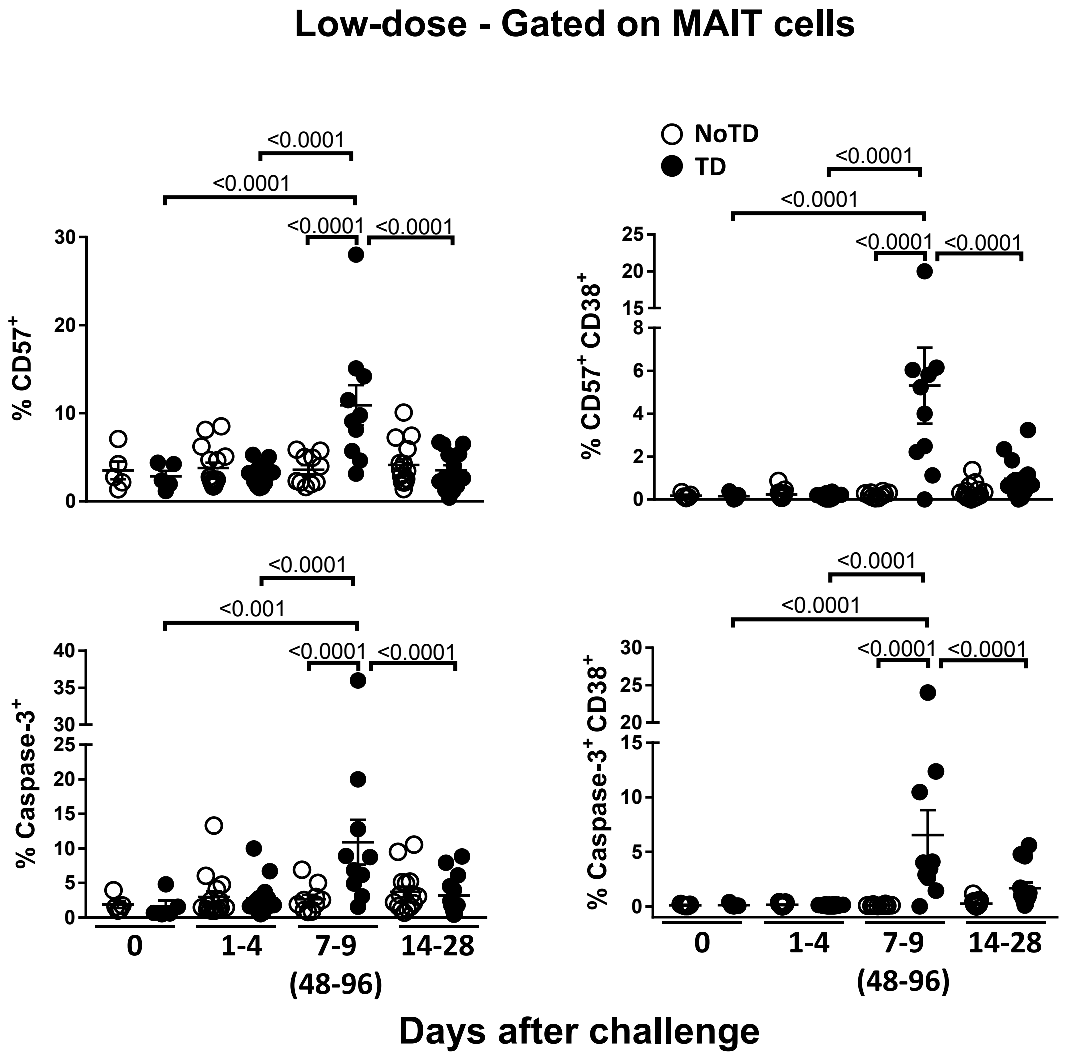


**Supplementary Figure 8. Comparison of exhaustion and apoptosis markers on MAIT cells among volunteers receiving a low-dose inoculum**. *Ex vivo* PBMC isolated before (Day 0, baseline) and after challenge were analyzed as described in Figure 1. Levels of (A) exhaustion (CD57) or (B) apoptosis (Caspase-3) on total or activated MAIT cells were evaluated and compared among NoTD and TD volunteers who received a low-dose inoculum. Data was grouped by time frame as follows: day 0, days 1-4, days 7-9 (for NoTD, or 48-96 hours for TD volunteers) and days 14-28. NoTD, individuals who did not develop typhoid disease. TD, Individuals who developed typhoid disease. *P* values of <0.05 were considered statistically significant.

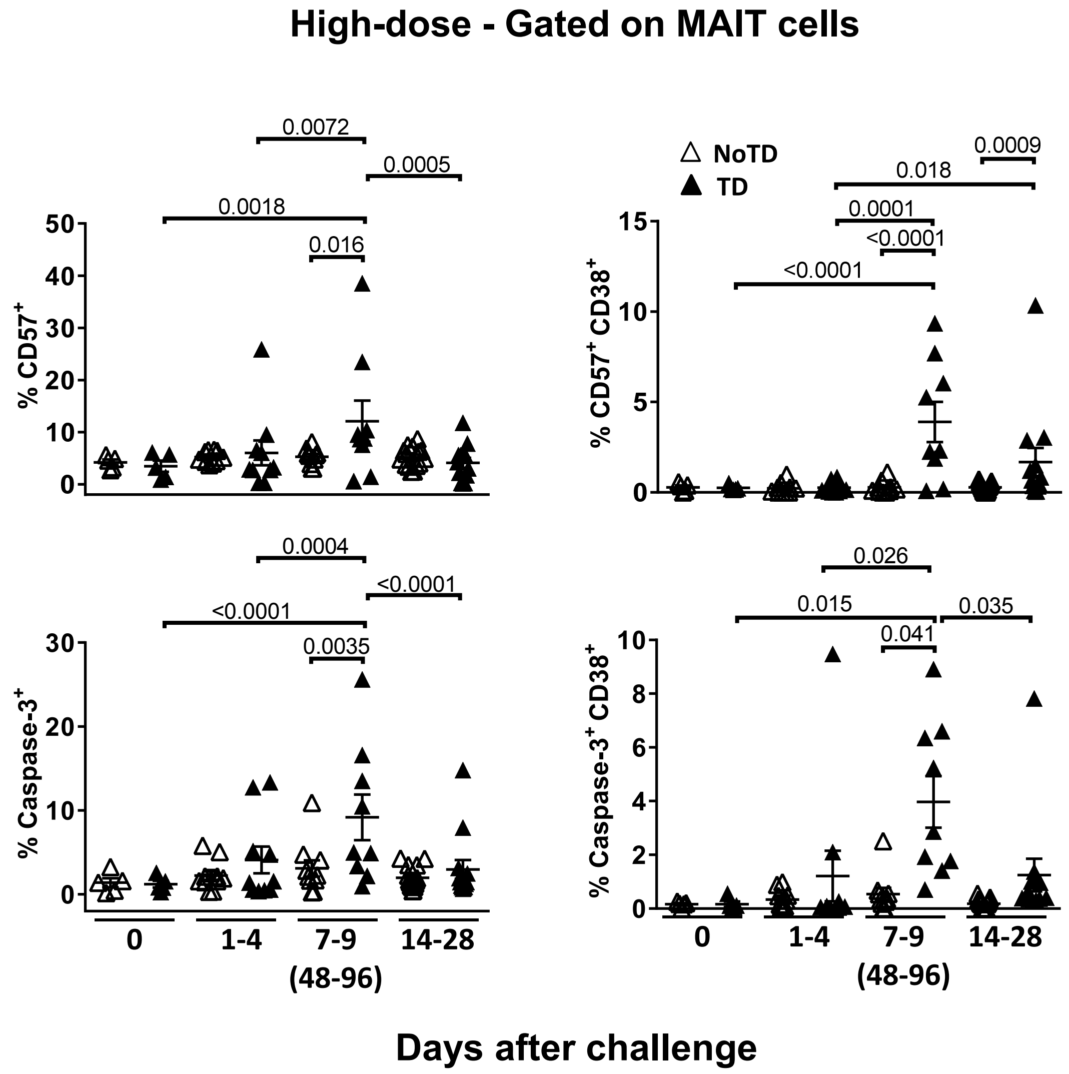


**Supplementary Figure 9. Comparison of exhaustion and apoptosis markers on MAIT cells among volunteers receiving a high-dose inoculum**. *Ex vivo* PBMC isolated before (Day 0, baseline) and after challenge were analyzed as described in Figure 1. Levels of (A) exhaustion (CD57) or (B) apoptosis (Caspase-3) on total or activated MAIT cells were evaluated and compared among NoTD and TD volunteers who received a high-dose inoculum. Data was grouped by time frame as follows: day 0, days 1-4, days 7-9 (for NoTD, or 48-96 hours for TD volunteers) and days 14-28. NoTD, individuals who did not develop typhoid disease. TD, Individuals who developed typhoid disease. *P* values of <0.05 were considered statistically significant.

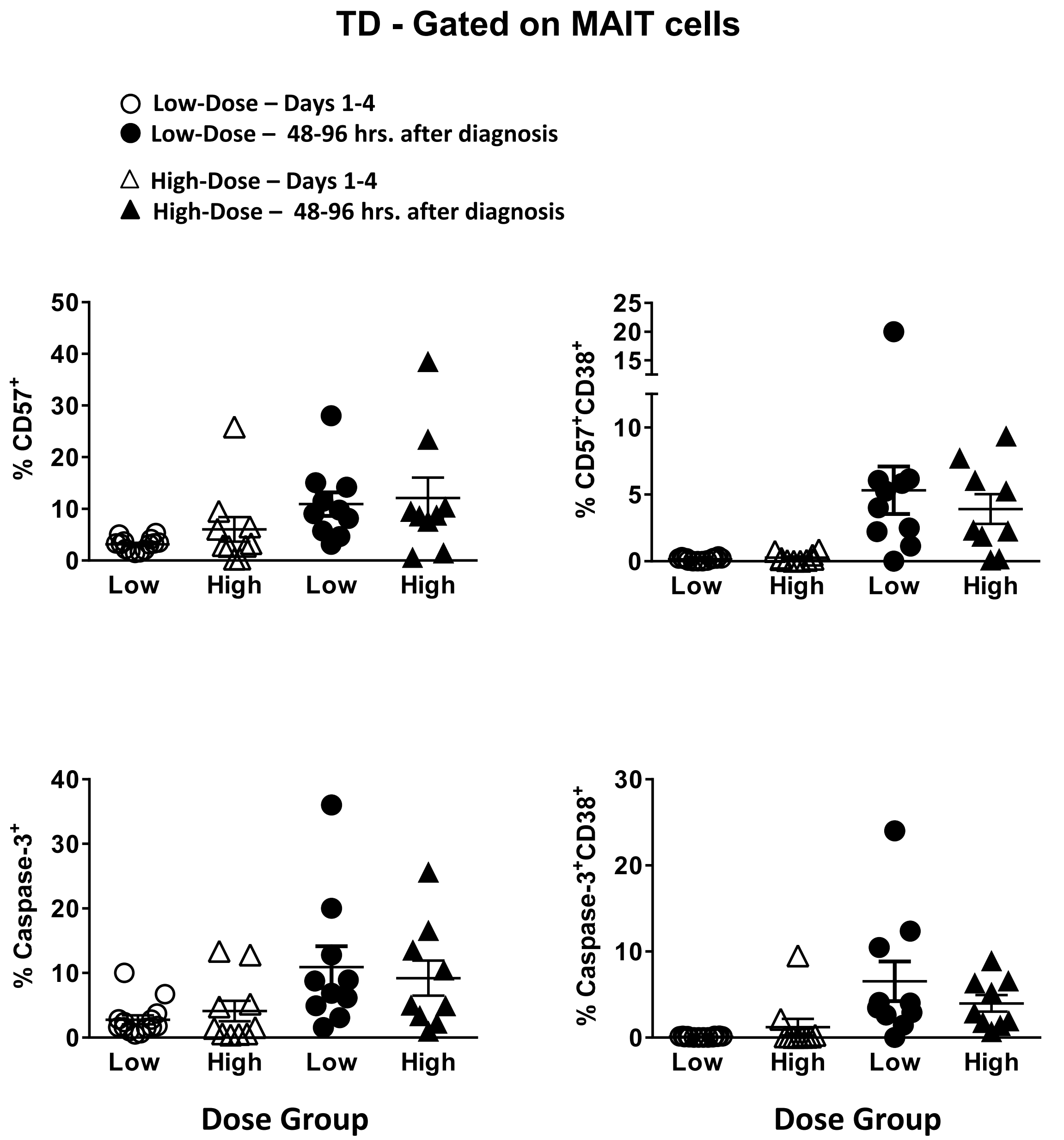


**Supplementary Figure 10. Comparison of the exhaustion and apoptosis markers on MAIT cells among TD volunteers receiving a high or low-dose inoculum**. *Ex vivo* PBMC isolated before (Day 0, baseline) and after challenge were analyzed as described in Figure 1. Levels of (A) exhaustion (CD57) or (B) apoptosis (Caspase-3) on total or activated MAIT cells were evaluated and compared among TD volunteers receiving a high or low-dose inoculum. Data was grouped by time frame as follows: before (days 1-4) and after development of disease (48-96 hours after diagnosis). TD, Individuals who developed typhoid disease.

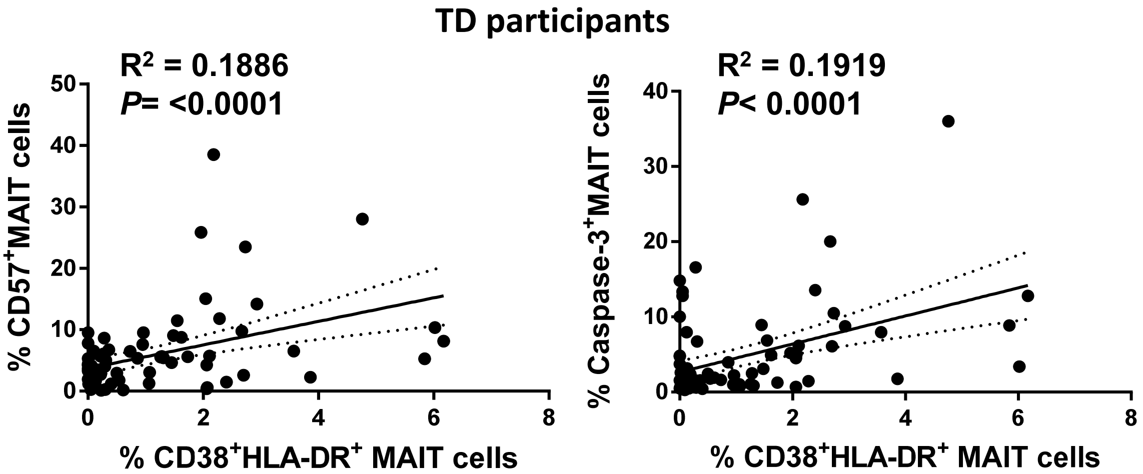


**Supplementary Figure 11. Direct correlation between MAIT cell activation and MAIT cell exhaustion and apoptosis**. *Ex vivo* PBMC were analyzed as described in Figures 1 & 2. The level of activated MAIT cells (CD38+HLA-DR+) was correlated to the levels of MAIT cells expressing either CD57 or caspase-3 surface markers. MAIT cells from samples of TD individuals were collected before and up to 28 days after challenge. TD, Individuals who developed typhoid disease. Coefficients of determination "R2" and "*P*" values are shown. *P* values of <0.05 were considered statistically significant.

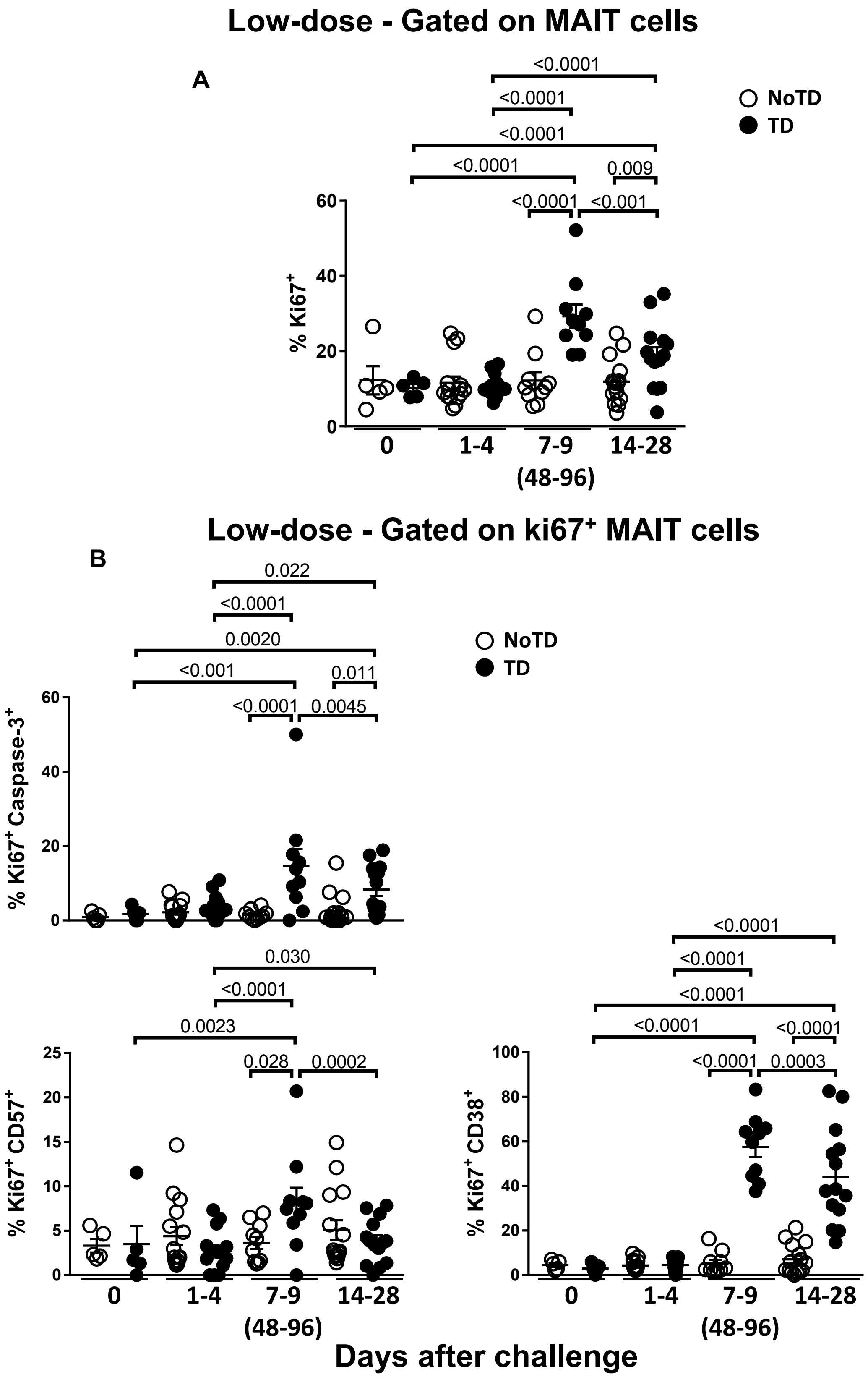


**Supplementary Figure 12. Comparison of the exhaustion and apoptosis markers on proliferating MAIT cells among volunteers receiving a low-dose inoculum**. *Ex vivo* PBMC isolated before (Day 0, baseline) and after challenge were analyzed as described in Figure 1. Levels of (A) total proliferating MAIT cells or (B) proliferating MAIT cells co-expressing CD38, CD57 or Caspase-3 surface markers were evaluated and compared among NoTD and TD volunteers who received a low-dose inoculum. Data was grouped by time frame as follows: day 0, days 1-4, days 7-9 (for NoTD, or 48-96 hours for TD volunteers) and days 14-28. NoTD, individuals who did not develop typhoid disease. TD, Individuals who developed typhoid disease. *P* values of <0.05 were considered statistically significant.

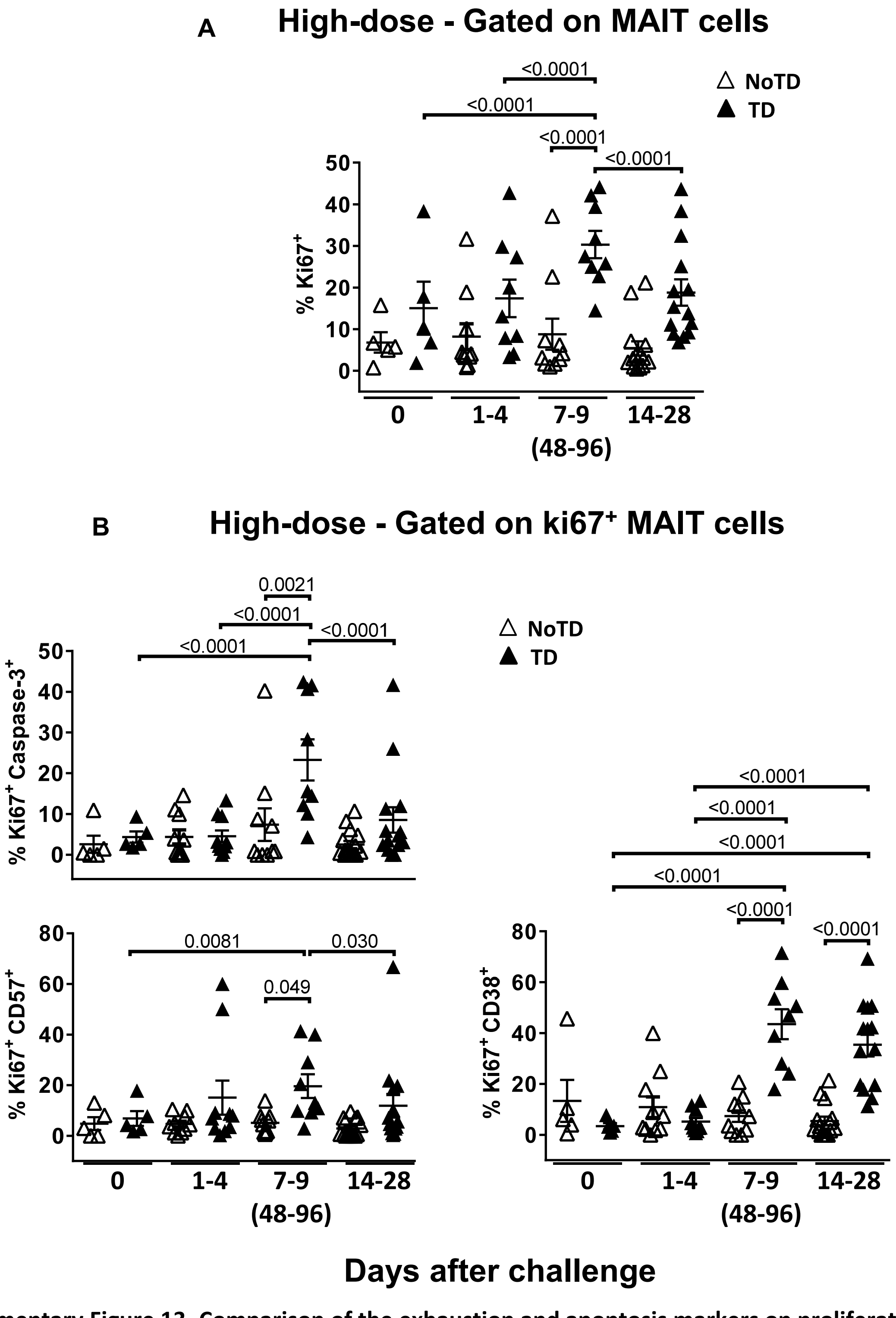


**Supplementary Figure 13. Comparison of the exhaustion and apoptosis markers on proliferating MAIT cells among volunteers receiving a high-dose inoculum**. *Ex vivo* PBMC isolated before (Day 0, baseline) and after challenge were analyzed as described in Figure 1. Levels of (A) total proliferating MAIT cells or (B) proliferating MAIT cells co-expressing CD38, CD57 or Caspase-3 surface markers were evaluated and compared among NoTD and TD volunteers who received a high-dose inoculum. Data was group by time frame as follows: day 0, days 1-4, days 7-9 (for NoTD, or 48-96 hours for TD volunteers) and days 14-28. NoTD, individuals who did not develop typhoid disease. TD, Individuals who developed typhoid disease. *P* values of <0.05 were considered statistically significant.

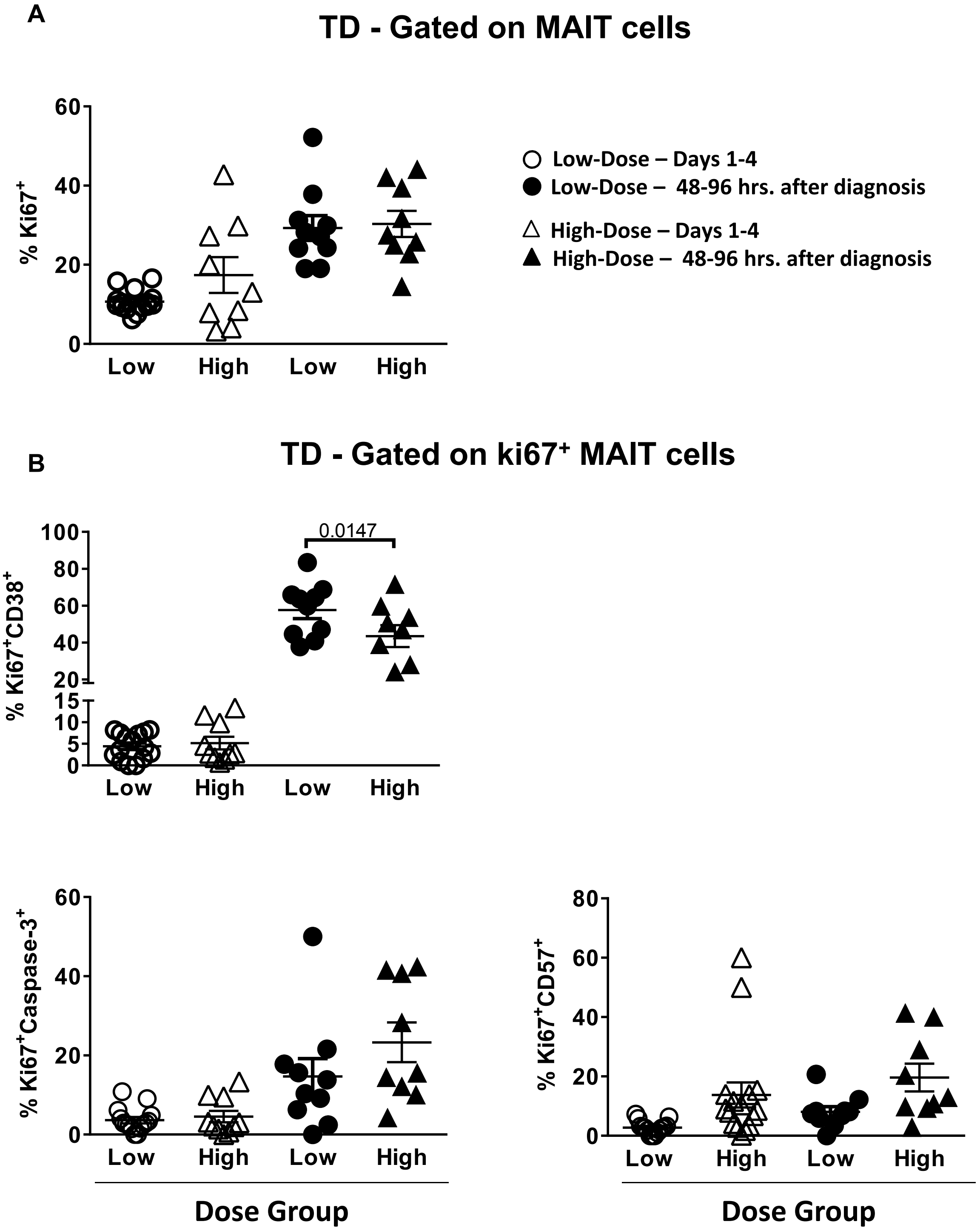


**Supplementary Figure 14. Comparison of the exhaustion and apoptosis markers on proliferating MAIT cells among TD volunteers receiving a low or high-dose inoculum**. *Ex vivo* PBMC isolated before (Day 0, baseline) and after challenge were analyzed as described in Figure 1. Levels of (A) total proliferating MAIT cells or (B) proliferating MAIT cells co-expressing CD38, CD57 or Caspase-3 surface markers were evaluated and compared among TD volunteers receiving high and low-dose inoculums. Data was grouped by time frame as follows: before (days 1-4) and after development of disease (48-96 hours after diagnosis). NoTD, individuals who did not develop typhoid disease. TD, Individuals who developed typhoid disease. *P* values of <0.05 were considered statistically significant.

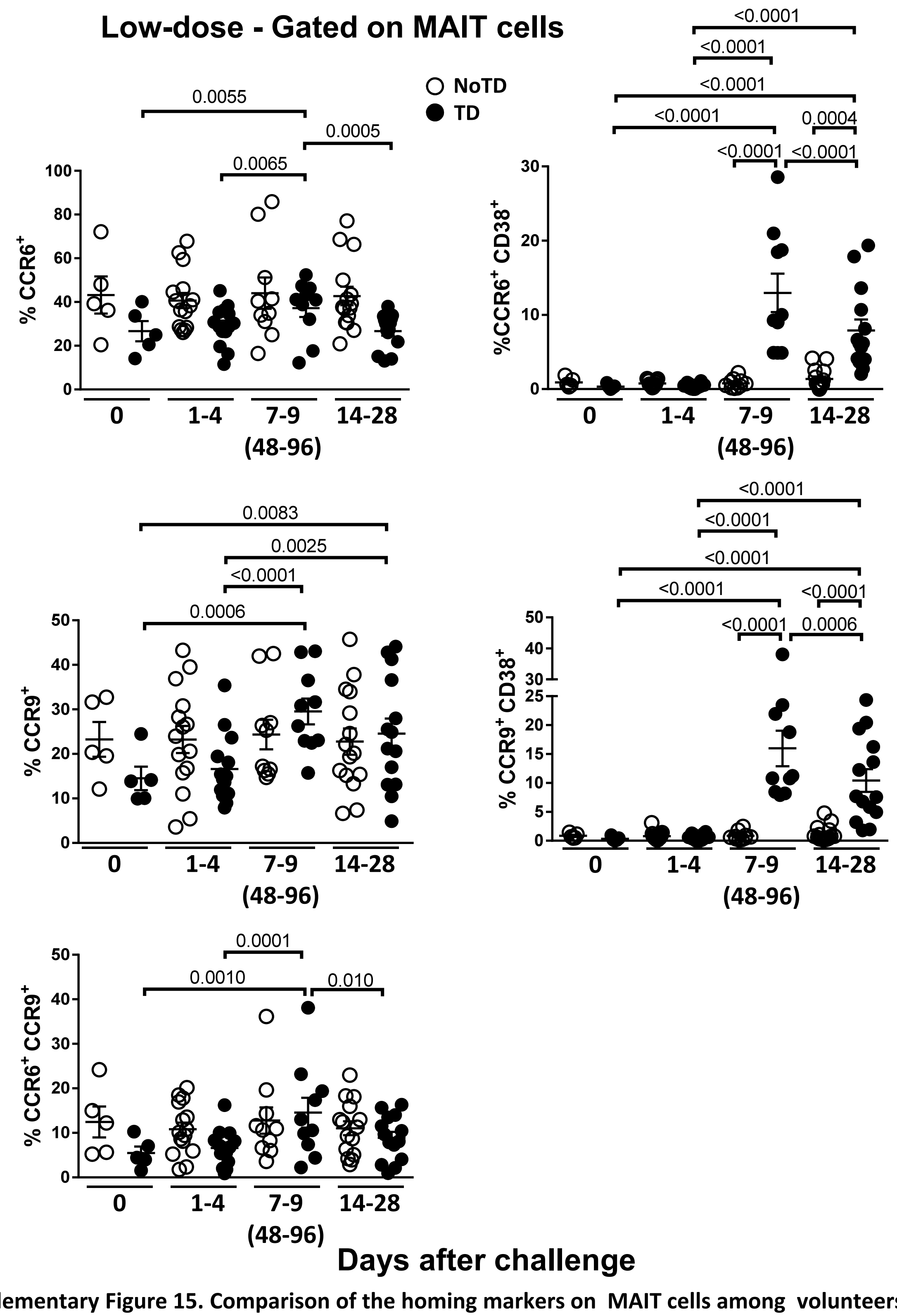


**Supplementary Figure 15. Comparison of the homing markers on MAIT cells among volunteers receiving low-dose inoculum**. *Ex vivo* PBMC isolated before (Day 0, baseline) and after challenge were analyzed as described in Figure 1. Additional gating on CCR6 and CCR9 were performed to identify MAIT cells with the potential to home to inflamed (CCR6) and gut (CCR9) tissues, respectively. Combined data of MAIT cells single expressing CCR6 or CCR9, or co-expressing CCR6 and CD38, CCR9 and CD38, CCR6 and CCR9 were compared among NoTD and TD volunteers who received a low-dose inoculum. Data was group by time frame as follows: day 0, days 1-4, days 7-9 (for NoTD, or 48-96 hours for TD volunteers) and days 14-28. NoTD, individuals who did not develop typhoid disease. TD, Individuals who developed typhoid disease. *P* values of <0.05 were considered statistically significant.

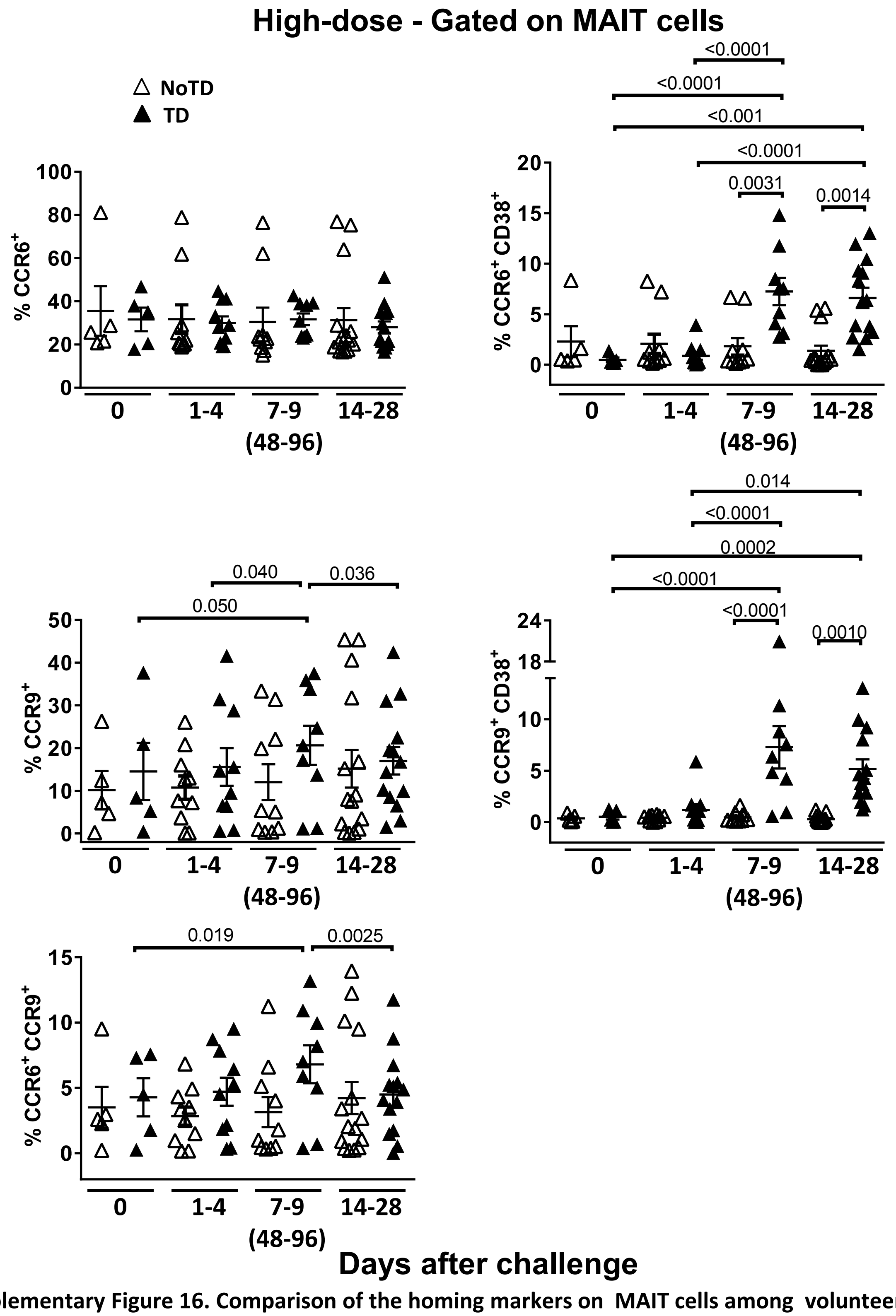


**Supplementary Figure 16. Comparison of the homing markers on MAIT cells among volunteers receiving a high-dose inoculum**. *Ex vivo* PBMC isolated before (Day 0, baseline) and after challenge were analyzed as described in Figure 1. Additional gating on CCR6 and CCR9 were performed to identify MAIT cells with the potential to home to inflamed (CCR6) and gut (CCR9) tissues, respectively. Combined data of MAIT cells single expressing CCR6 or CCR9, or co-expressing CCR6 and CD38, CCR9 and CD38, CCR6 and CCR9 were compared among NoTD and TD volunteers who received a high-dose inoculum. Data was grouped by time frame as follows: day 0, days 1-4, days 7-9 (for NoTD, or 48-96 hours for TD volunteers) and days 14-28. NoTD, individuals who did not develop typhoid disease. TD, Individuals who developed typhoid disease. *P* values of <0.05 were considered statistically significant.

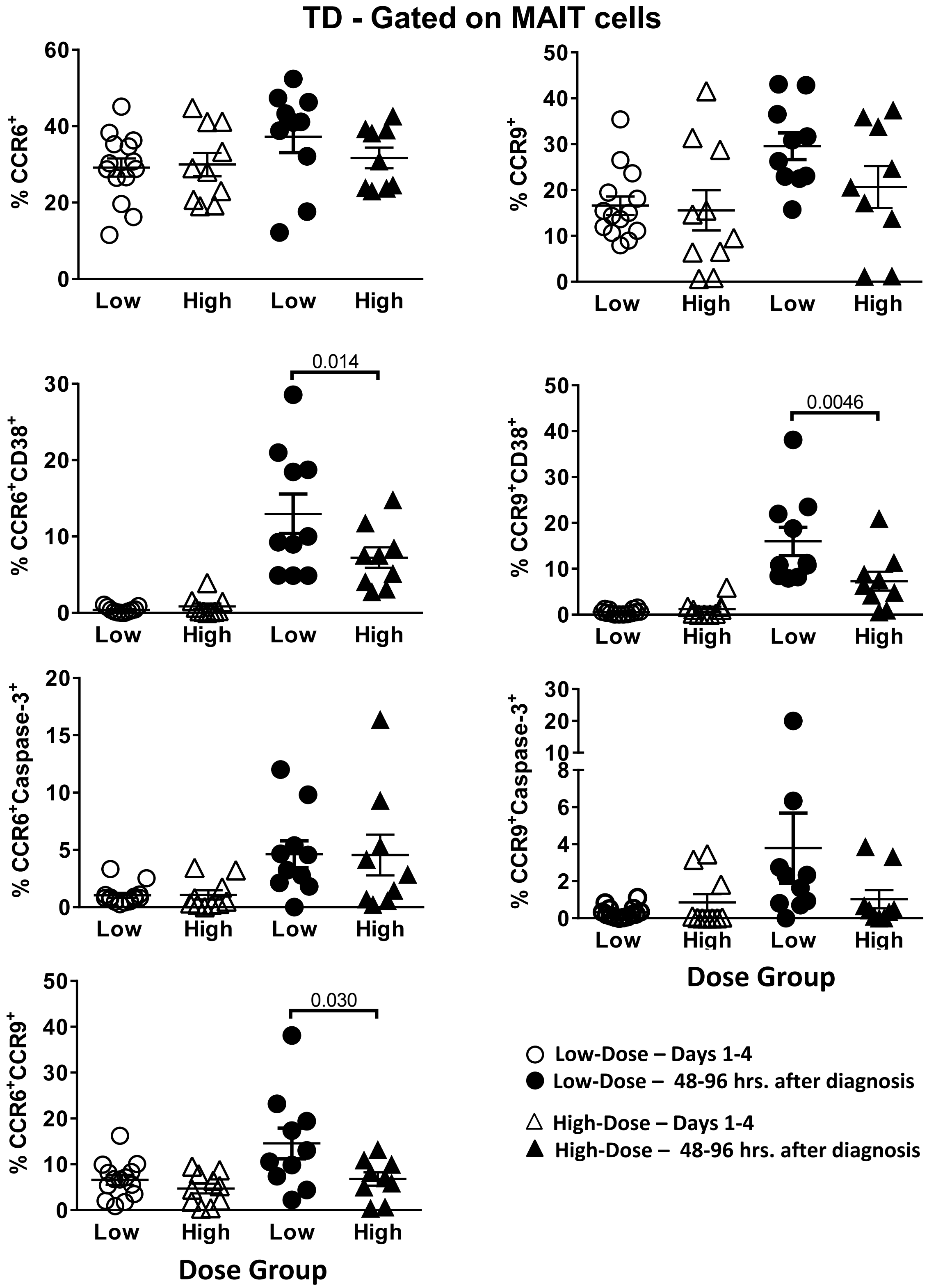


**Supplementary Figure 17. Comparison of the homing markers on MAIT cells among volunteers receiving a high- or low-dose inoculum who developed TD**. *Ex vivo* PBMC isolated before (Day 0, baseline) and after challenge were analyzed as described in Figure 1. Additional gating on CCR6 and CCR9 were performed to identify MAIT cells with the potential to home to inflamed (CCR6) and gut (CCR9) tissues, respectively. Combined data of MAIT cells single expressing CCR6 or CCR9, or co-expressing CCR6 and CD38, CCR9 and CD38, CCR6 and CCR9, CCR6 and Caspase-3, or CCR9 and Caspase-3 were evaluated and compared among TD volunteers receiving a low or high-dose inoculum. Data was grouped by time frame as follows: before (days 1-4) and after development of disease (48-96 hours after diagnosis). TD, Individuals who developed typhoid disease. *P* values of <0.05 were considered statistically significant.

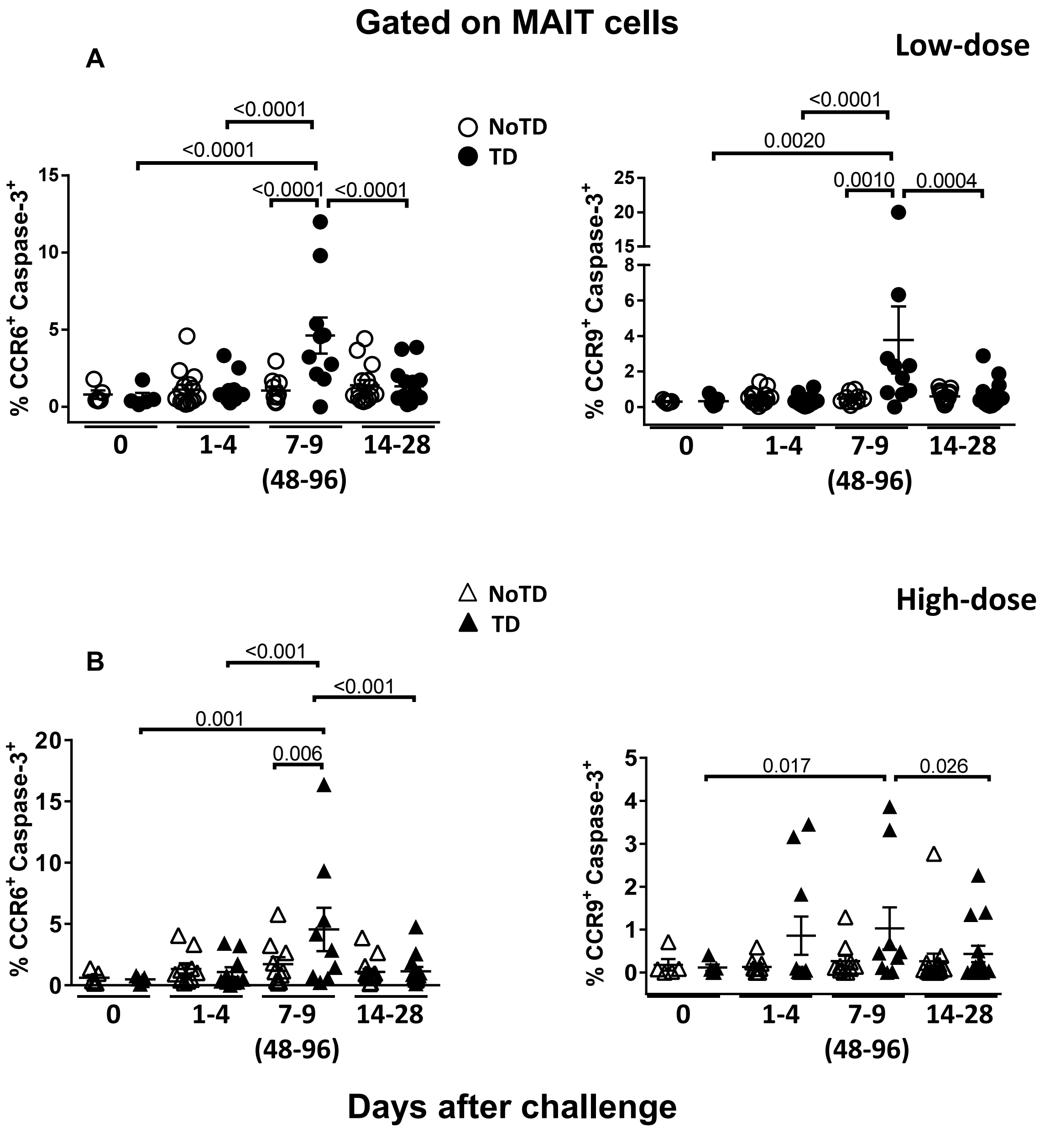


**Supplementary Figure 18. Comparison of the homing markers on MAIT cells among volunteers receiving a low or high-dose inoculum**. *Ex vivo* PBMC isolated before (Day 0, baseline) and after challenge were analyzed as described in Figure 1. Additional gating on CCR6 and CCR9 were performed to identify MAIT cells with the potential to home to inflamed (CCR6) and gut (CCR9) tissues, respectively. Combined data of MAIT cells co-expressing CCR6 and Caspase-3, or CCR9 and Caspase-3 were evaluated and compared among NoTD and TD volunteers receiving a low- (A) or high-dose (B) inoculum. Data was grouped by time frame as follows: before (days 1-4) and after development of disease (48-96 hours after diagnosis). NoTD, individuals who did not develop typhoid disease. TD, Individuals who developed typhoid disease. *P* values of <0.05 were considered statistically significant.

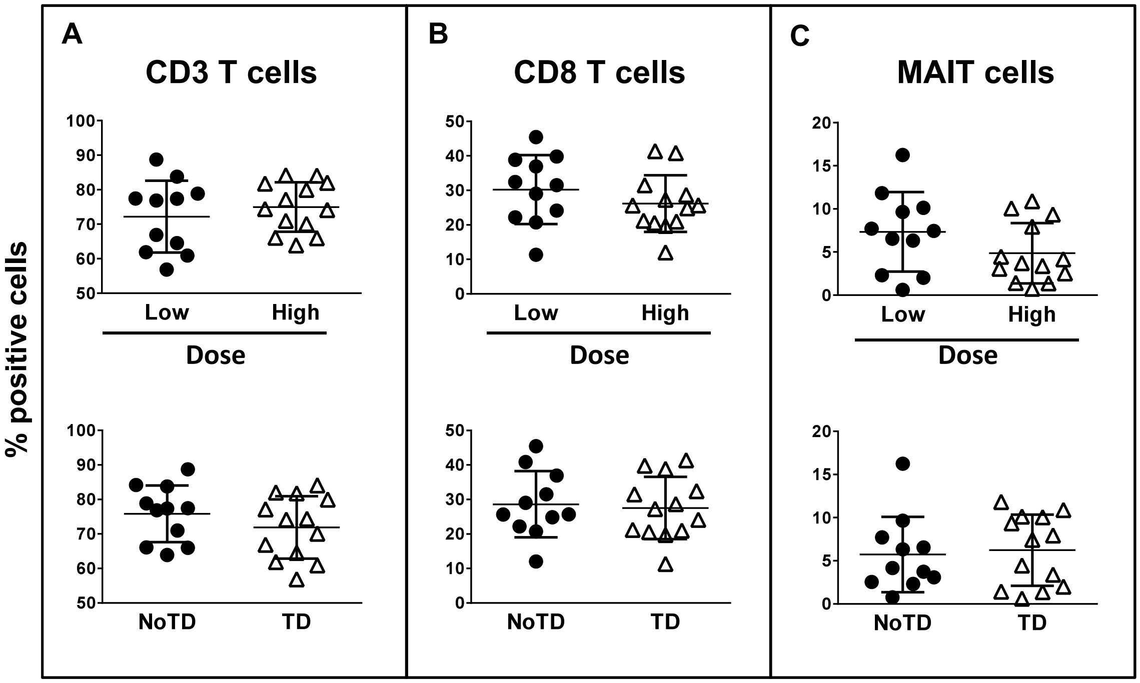


**Supplementary Figure 19. Baseline levels of CD3, CD8 and MAIT cells**. *Ex vivo* PBMC isolated before challenge (Day 0, baseline) were analyzed as described in Figure 1. Levels of (A) CD3, (B) CD8, and (C) MAIT cells were evaluated and compared among groups. Lines represent mean± SD of each group. NoTD, individuals who did not develop typhoid disease. TD, Individuals who developed typhoid disease
